# Comprehensive Study of Li Deposition and Solid Electrolyte Cracking by Integrating Simulation and Experimental Data

**DOI:** 10.1002/advs.202501434

**Published:** 2025-03-16

**Authors:** Chen Lin, Haihui Ruan, Ming‐Sheng Wang

**Affiliations:** ^1^ Sino‐French Institute of Nuclear Engineering and Technology Sun Yat‐Sen University Zhuhai 519000 China; ^2^ Department of Mechanical Engineering The Hong Kong Polytechnic University Hung Hom Kowloon Hong Kong 999077 China; ^3^ State Key Laboratory of Physical Chemistry of Solid Surfaces College of Materials Xiamen University Xiamen 361000 China

**Keywords:** cracking, deposition, integrating simulation and experimental data, mechanical constraint

## Abstract

Lithium (Li) penetration into solid‐state electrolytes (SE) is a major cause of lithium‐metal solid‐state battery (LMSSB) failure. However, no single model fully explains experimental phenomena, and many simulation‐based conclusions lack validation or contradict experimental results, hindering the understanding of failure mechanisms. This study integrates simulation and experimental data to investigate Li deposition and SE cracking, introducing a unified phase‐field (PF) model. Unlike existing models, it accounts for mechanical constraints, solid–solid contact, and large‐strain mechano‐chemical coupling. It also distinguishes Li penetration from SE cracking, as short‐circuiting and cracking do not occur simultaneously. Additionally, crack initiation follows the pressurized cracking model, while propagation occurs through a wedge‐shaped opening. A counterintuitive approach to extending LMSSB lifespan is to reduce the mechanical constraints of SE rather than decreasing defect size or increasing SE hardness and toughness, provided that good contact is maintained between the electrode and SE. This is because minimizing mechanical constraints alters the Li deposition mode, preventing rapid Li eruption in cracks.

## Graphical abstract

This study presents a phase‐field model integrating mechanical constraints, solid–solid contact, and large‐strain mechanochemical coupling to characterize lithium deposition and electrolyte cracking. Results show that reducing mechanical constraints, with good electrode–electrolyte contact, suppresses dendrite eruption in cracks and extends battery lifespan, providing new insights for optimizing lithium‐metal solid‐state batteries.

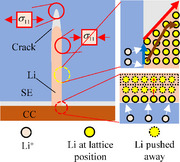

## 1 Introduction

The rapid development of human society has led to a critical challenge: energy shortages. Global energy demand is projected to reach 28–35 TW by 2050.^[^ ^1^ ^]^ To address this issue, there is an urgent need to transition the global energy landscape toward renewable sources. However, most renewable energy sources, including wind, solar, tidal, biomass, and geothermal energy, exhibit uneven spatial and temporal distributions. This underscores the necessity of developing advanced energy storage technologies to optimize energy structures, promote environmentally sustainable development, and enhance overall energy utilization efficiency.

Lithium‐ion batteries (LIBs) are widely regarded as the leading electrochemical energy storage technology due to their high energy and power density, excellent cyclability, and reliability. However, LIBs still face challenges, including the insufficient theoretical capacity of both positive and negative electrodes, as well as safety concerns.^[^ ^3^ ^]^ Lithium‐metal solid‐state batteries (LMSSBs) address some of these limitations by replacing the flammable liquid electrolyte in conventional LIBs with a nonflammable solid electrolyte, thereby enhancing thermal stability and reducing the risk of battery fires and explosions under extreme conditions. Furthermore, LMSSBs employ lithium (Li) metal instead of graphite as the anode, significantly increasing theoretical capacity density. Additionally, LMSSBs can rapidly accommodate and release Li ions, enabling fast charging without electrolyte polarization. Consequently, LMSSBs are considered the “holy grail” of next‐generation high‐energy‐density lithium‐metal batteries (LMBs) and have attracted widespread attention.^[^ ^4^, ^5^ ^]^

However, contrary to expectations, the performance of LMSSBs has been observed to degrade significantly or even fail after several charge–discharge cycles. The primary cause of this deterioration and failure is the uncontrolled penetration of Li dendrites during the charging process.^[^ ^6^, ^7^, ^8^ ^]^ Two decades ago, Monroe and Newman^[^ ^9^, ^10^ ^]^ proposed that if the shear modulus of an organic SE exceeds twice that of Li metal, it can suppress the penetration of Li dendrites. This led to the prevailing belief that inorganic SE, which generally exhibit higher shear moduli than organic SE, should be capable of preventing Li dendrite penetration, thereby driving the development of inorganic SE. However, despite their high hardness and toughness, inorganic SE have been unable to prevent Li dendrite penetration,^[^ ^11^, ^12^ ^]^ contradicting Monroe and Newman's theoretical predictions.

A key reason for this discrepancy is that the mechanism of Li dendrite formation and growth in inorganic SE differs significantly from that in organic SE. In liquid or organic SE, Li dendrite formation and growth result from amplified kinetic perturbations at the Li‐electrolyte interface, and increasing the electrolyte shear modulus can effectively suppress these perturbations. In contrast, in inorganic SE, pre‐existing defects serve as the primary sites for Li dendrite formation, while crack propagation from these defects is the main factor driving dendrite growth.

To explain Li dendrite penetration in SE, the well‐known pressurized cracking model^[^ ^13^ ^]^ was proposed. According to this model, Li preferentially deposits at pre‐existing defects. Once these defects are filled, continued charging forces the excess deposited Li to exert pressure on the defect sidewalls, leading to SE cracking. Since Li possesses a lower modulus of elasticity and yield strength than SE, the model assumes that Li behaves similarly to a fluid. This “flowing” Li can immediately fill the new space created by the crack, further driving crack propagation. Ultimately, the simultaneous expansion of cracks and Li dendrite penetration leads to the failure of LMSSBs.

In a recent study, Hao et al.^[^ ^14^ ^]^ employed in situ X‐ray computed tomography and spatial mapping X‐ray diffraction to monitor Li penetration and crack propagation in LMSSBs. Their findings revealed that Li dendrite penetration and crack propagation do not occur simultaneously. Instead, crack propagation occurs at a much faster rate than Li dendrite penetration, indicating that cracking does not immediately induce a short circuit in LMSSBs.

Additionally, Ning et al.^[^ ^15^, ^16^ ^]^ identified two distinct stages in Li dendrite penetration: dendrite formation and dendrite propagation. Dendrite formation follows the pressurized cracking model,^[^ ^13^ ^]^ in which Li fills the defect, leading to cracking. The localized fracture strength at the crack site is correlated with the defect size. Dendrite propagation, on the other hand, occurs through a wedge‐shaped opening mechanism,^[^ ^16^ ^]^ wherein Li accumulates at the bottom of the crack, while the crack tip remains Li‐free. The Li at the crack base exerts compressive stress, driving the extension of the crack tip. The fracture toughness at the crack tip aligns with the macroscopic fracture toughness of the SE. Furthermore, experimental studies have demonstrated that mechanical constraints within the LMSSB system have a significant influence on Li deposition. For instance, Ning et al.^[^ ^16^ ^]^ observed that at a stack pressure of 7 MPa, LMSSBs failed after 35 cycles. In contrast, when subjected to atmospheric pressure (0.1 MPa), the lifespan of LMSSBs extended to 170 cycles.

Understanding Li deposition and SE cracking is influenced by multiple physical factors. However, fully capturing these physical processes through experiments alone remains challenging. Additionally, real‐time observation of dynamic changes occurring beneath the SE surface is difficult. Consequently, multiphysics‐based modeling has become essential to supplement experimental findings and elucidate the mechanisms underlying Li deposition and SE cracking.

Tantratian et al.^[^ ^17^ ^]^ employed density functional theory (DFT) calculations and phase‐field (PF) methods to investigate the influence of surface electronic states and grain size on Li dendrite penetration. Their simulations demonstrated Li nucleation at SE grain boundaries, followed by the penetration and interconnection of Li filaments, ultimately leading to a short circuit in LMSSBs. However, their model did not treat Li penetration and SE cracking as independent processes. Similar simplifications were adopted by Yuan et al.^[^ ^18^, ^19^ ^]^ and Lin et al.,^[^ ^20^ ^]^ where crack extension and dendrite penetration were modeled to occur along the same path and at the same rate—an assumption that contradicts experimental observations.^[^ ^14^ ^]^ Liu et al.,^[^ ^21^ ^]^ Xu et al.,^[^ ^22^ ^]^ and Xiong et al.^[^ ^23^ ^]^ investigated SE cracking induced by deposited Li, assuming that Li was initially present within pre‐existing defects. Their model applied a volume expansion associated with the deposition reaction to simulate Li extrusion along the defect sidewalls, while cracking was modeled using damage mechanics. However, their approach did not account for the continued penetration of Li following crack formation. As a result, their model may be more suitable for describing crack initiation rather than explaining how deposited Li drives crack extension, as defined by Ning et al.^[^ ^16^ ^]^ Furthermore, Fang et al.^[^ ^24^ ^]^ examined the effect of stacking pressure on Li penetration within voids by incorporating an advection term into the Allen–Cahn equation within the phase‐field model. However, their study did not address the modeling of the cracking process.

Modeling Li permeation and SE cracking is generally considered as two relatively independent processes. Lin et al.^[^ ^24b^ ^]^ and Monismith et al.^[^ ^25^ ^]^ developed similar PF models to investigate LMSSB failure during charging. In their models, the fracture PF method was used to simulate crack initiation and propagation, providing insights into Li dendrite penetration in SE, including dendrite initiation, crack formation, and the driving of crack extension by deposited Li. However, their approach to simulating dendrite formation relies on kinetic perturbations, which are more applicable to liquid Li batteries rather than LMSSBs. Additionally, their models represent cracking by artificially introducing either simple plating stress or Li volume expansion, which does not accurately capture the solid–solid contact between deposited Li and the defective sidewalls. Furthermore, determining the magnitude of plating stress or volume expansion remains controversial and ambiguous in many studies.^[^ ^18^, ^19^, ^20^, ^21^, ^22^, ^23^, ^24^, ^25^ ^]^

At present, there is a need for more comprehensive theoretical models capable of fully explaining experimentally observed phenomena. Moreover, many numerical studies^[^ ^18^ ^–^ ^24b^ ^]^ remain qualitative and lack direct comparison with experimental results. This discrepancy suggests that conclusions drawn from numerical simulations are often unverified or contradicted by experimental findings. To achieve a thorough and precise understanding of Li deposition and electrolyte cracking during charging, it is essential to integrate experimental data with modeling in a systematic and comprehensive manner.

To conduct a comprehensive study integrating experimental data and modeling, it is essential to observe Li deposition and SE cracking through high‐temporal and high‐spatial‐resolution in situ experiments. Gao et al.^[^ ^26^ ^]^ successfully captured a series of mechano‐chemical behaviors during the charging process of a sub micrometer single‐crystal SE using high‐resolution transmission electron microscopy. Notably, selecting a single‐crystal SE eliminates the influence of grain boundaries, facilitating the quantitative tracking of Li penetration and electrolyte cracking. Their study revealed that under weak mechanical constraints between the electrode and SE, Li tends to deposit perpendicularly to the SE surface in the form of whiskers, while simultaneously pushing the electrode away from the SE in a process known as root growth.^[^ ^27^, ^28^, ^29^ ^]^ When the mechanical constraint reaches a specific threshold, root growth ceases, and the whisker shanks begin to expand transversely. Further increasing the mechanical constraint leads to SE cracking and Li infiltration. Additionally, Gao et al.’s experiments demonstrated that when single‐crystal SE sidewalls are constrained by neighboring grains, Li penetration, and crack propagation occur almost simultaneously, resulting in a short‐circuit failure in a very short time. Conversely, in an isolated single‐crystal SE without grain constraints, a significantly longer time elapses between crack formation and Li penetration, thereby delaying the onset of short‐circuit failure.

There is an urgent need for a unified theoretical model to explain these experimentally observed phenomena. To address this, we propose a unified model based on the PF approach to elucidate the complex mechano‐chemical coupling observed in experiments. Unlike existing models,^[^ ^18^, ^19^, ^20^, ^21^, ^22^, ^23^, ^24^, ^25^ ^]^ our model explicitly incorporates the effect of mechanical constraints on Li deposition modes. Under low mechanical constraints, Li deposition on the electrolyte surface displaces the original host lattice, a process known as root growth. In contrast, under high mechanical constraints, Li fills defects or the electrolyte–electrode gap through atomic diffusion (also referred to as dislocation climbing), a process described as the viscoplastic flow mode. Building on our previous study,^[^ ^24b^ ^]^ our model treats Li penetration and SE cracking as two relatively independent processes. A key distinction is that the stresses arise from the solid–solid contact between deposited Li and crack sidewalls, rather than being artificially imposed through electroplating stresses or volume expansion. To simulate solid–solid contact within a continuous medium framework, we assign minimal stiffness in the tangential direction along the Li‐electrolyte interface. This allows Li to flow relative to the sidewalls without generating unrealistic shear stresses. The model describes crack propagation in two stages: initiation and extension, employing the pressurized cracking model^[^ ^13^ ^]^ and the wedge opening model,^[^ ^15^, ^16^ ^]^ respectively. The constitutive relationship is established within the large‐strain framework, as stress‐induced cracking reaches the GPa level. The large‐strain theory incorporates higher‐order terms of geometric nonlinearity, allowing for a more accurate representation of significant deformations and stresses. Additionally, a coupled mechano‐chemical electroplating kinetic equation was developed within the large‐strain framework. This equation integrates the stress‐dependent term of the electroplating reaction in the form of the Eshelby stress tensor, thereby simultaneously accounting for the effects of stress and conformational volume changes on the electroplating reaction.

To conduct a comprehensive study, we compare simulation results with experimental data. This includes analyzing the height and volume of Li whiskers deposited on the electrolyte surface, the volume of Li dendrites penetrating the SE, and the deposition current density. These comparisons will allow us to validate the model's accuracy from multiple perspectives. Building on this foundation, we aim to develop a thorough understanding of Li deposition and SE cracking, as well as to investigate the influence of mechanical constraints on Li deposition modes and LMSSB failure. Additionally, we seek to uncover the complex mechano‐chemical coupling mechanisms governing the charging process. Ultimately, our objective is to propose an effective strategy for extending the lifespan of LMSSBs.

## 2 Methodology

### 2.1 Distribution of Multiphase Field and Crack

**Figure 1:**
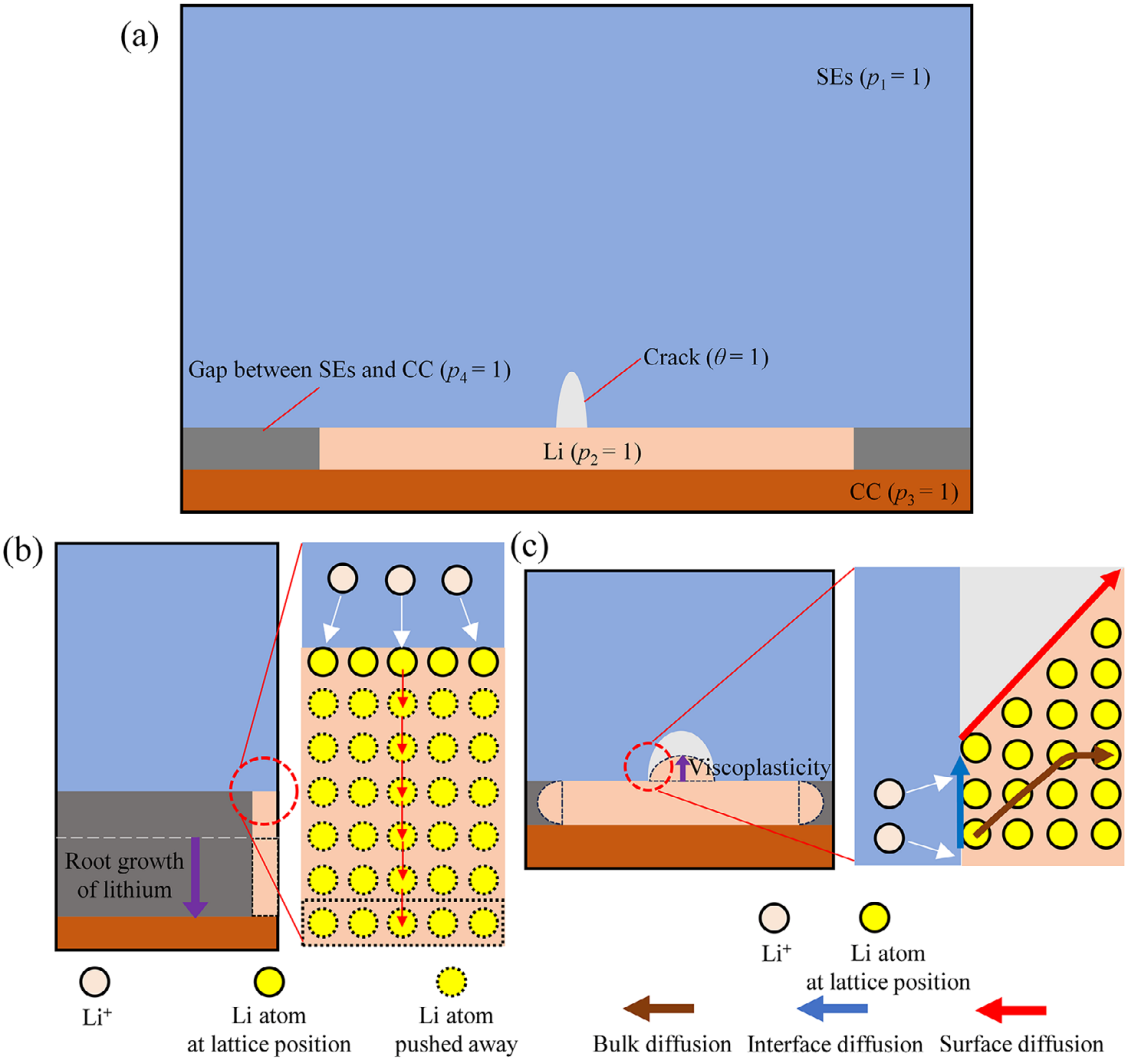
Schematic diagram of the spatial distribution of order parameter field, **p**, and fracture order parameter, *θ*.

This paper presents a theoretical framework for a Li plating scenario, as illustrated in **Figure** **1**. In this scenario, a layer of Li is plated onto a SE with pre‐existing flaws, leading to the formation of gaps between the SE and the current collector (CC). According to Gao et al.,^[^ ^26^ ^]^ the root‐growth mechanism^[^ ^27^, ^28^, ^29^ ^]^ occurs under weak mechanical constraint conditions. In this case, the deposited Li displaces the original host lattice, as depicted in Figure 1b. Conversely, under significant mechanical constraints, the deposited Li fills the cavities on the SE surface or the gaps between the SE and CC, as shown in Figure 1c.

To address this issue, we introduce a vector of order parameters, **p** = (*p*_1_, *p*_2_, *p*_3_, *p*_4_), along with the fracture order parameter, *θ*. As shown in Figure 1, the components *p*_1_, *p*_2_, *p*_3_, and *p*_4_ correspond to the SE, Li, CC, and gaps, respectively. Each phase is characterized by *p_i_* = 1 within its respective domain, smoothly transitioning to 0 across boundaries with other phases, while maintaining the condition $\sum_{i=1}^{N}p_{i}=1$ ^[^ ^30^ ^]^ among these phases. Cracks are represented by the order parameter *θ*, where *θ* = 1 in intact regions and *θ* = 0 in cracked regions. Cracks are treated separately, and for any physical properties across interfaces, interpolation functions $h_{\alpha }={\left(p_{\alpha }\right)}^{2}/\sum_{i=1}^{N}{\left(p_{i}\right)}^{2}$ ^[^ ^30^ ^]^ and χ(θ) = θ^3^(10 − 15θ + 6θ^2^)^[^ ^31^ ^]^ are employed to ensure smooth transitions, respectively.

We allow the order parameters *θ* and *p*_2_ to “overlap,” which represents a scenario where a crack and Li occupy the same physical space. Specifically, this means that a crack forms in the SE, while Li partially fills it. Therefore, in the theoretical model, there is a partial overlap between the crack and Li phases within this shared spatial region. However, the physical mechanisms governing Li growth and crack propagation are fundamentally distinct and must be described using separate governing equations. Nonetheless, these processes are interrelated, and their interaction must be appropriately accounted for. For instance, the model should ensure that Li metal deposits exclusively within cracks rather than other locations. Additionally, it must consider how crack formation influences the mechanical properties of the material.

### 2.2 Constitutive Relationship Under Large‐Strain Framework

The crack opening caused by Li insertion is a large‐deformation problem. Therefore, the following constitutive relation between the stress tensor **σ** and the deformation gradient **F =** ∂**x**/∂**X** (where **X** and **x** represent the coordinates of a material point before and after deformation, respectively) is adopted

(1) $$\sigma =\det{\left(F\right)}^{-1}\left(F^{e}\frac{\partial \Psi^{\mathrm{mech}}}{\partial E^{e}}{\left({\left(F^{\mathrm{vol}}\right)}^{T}{\left(F^{\mathrm{vp}}\right)}^{T}\right)}^{-1}\right)F^{T}$$

where **σ** is the Cauchy stress tensor; **F** is divided into **F** ^e^, **F** ^vp^, and **F** ^vol^ representing the elastic, viscoplastic, and volumetric deformation gradients, respectively; **E** ^e^ = (**F** ^e*T*^ **F** ^e^ − **I**)/2 is the Green–Lagrange elastic strain tensor; ψ^mech^ is elastic strain energy density per unit volume (note: herein the volume means the volume of the original state); det(•) indicates the determinant of a matrix.

The Li‐SE and Li‐CC interfaces result from solid–solid contact, allowing for relative sliding between the materials. In practice, interfacial friction or shear force is not negligible due to the adhesion between Li and SE. However, the limitations of current experimental techniques prevent the quantitative measurement of the friction coefficient or shear force at these interfaces. Consequently, in this model, the interface is idealized as frictionless. To address this simplification, a small tangential stiffness tensor is introduced along the Li surface to ensure that interfacial shear stresses at solid–solid interfaces remain minimal. This tangential stiffness tensor serves as a numerical approximation rather than an experimentally determined value. Such an approximation is necessary because the PF approach is based on the assumption of a continuous medium. Without this approximation, the continuous‐medium assumption would lead to an overestimation of interfacial shear stresses when modeling the mechanical behavior of discontinuous interfaces. By incorporating this approximation, the model effectively mitigates or reduces interfacial shear stresses within the continuous‐medium framework. Therefore, the mechanical strain energy ψ^mech^ is expressed as

(2) $$\psi^{\mathrm{mech}}=J^{\mathrm{vol}}\psi_{0}^{\mathrm{mech}}=J^{\mathrm{vol}}\left(\frac{1}{2}E^{e}:\left(C_{\mathrm{bulk}}^{e}+C_{\mathrm{contact}}^{e}\right):E^{e}\right)$$

where $\psi_{0}^{\mathrm{mech}}$ is the elastic strain energy per unit volume for a deformed geometry, which is converted to ψ^mech^ by multiplying *J* ^vol^ which is the volume change at a material point **X**. In Equation (2), $C_{\mathrm{bulk}}^{e}$ and $C_{\mathrm{contact}}^{e}$ denote the bulk and surface tangential stiffness tensors of Li, respectively, which are expressed as

(3) $$C_{\mathrm{bulk}}^{e}=h_{1}\left(\left(1-\chi \left(\theta \right)\right)+\eta \chi \left(\theta \right)\right)C_{1}^{e}+h_{2}C_{2}^{e}+h_{3}C_{3}^{e}+\eta h_{4}C_{1}^{e}$$

(4) $$\begin{array}{cc} C_{\mathrm{contact}}^{e} & =16{\left(p_{2}\right)}^{2}{\left(1-p_{2}\right)}^{2}T\eta C_{1}^{e}\\ & =16{\left(p_{2}\right)}^{2}{\left(1-p_{2}\right)}^{2}\left(I-\frac{1}{{\left|\nabla p_{2}\right|}^{2}}\nabla p_{2}⊗\nabla p_{2}\right)\eta C_{1}^{e} \end{array}$$

where $C_{1}^{e}$, $C_{2}^{e}$, $C_{3}^{e}$ are the stiffness tensors of the SE, Li, and CC, respectively. Note that the interpolation functions of *h_α_* and *χ*(*θ*) are used to mollify the discontinuity across different phases and cracks. In Equation (4), the term 16(*p*_2_)^2^(1−*p*_2_)^2^ ensures that the stiffness matrix, $C_{\mathrm{contact}}^{e}$, only signifies at the surface of Li deposit, **T** = **I** − (∇*p*_2_⊗∇*p*_2_)/|∇*p*_2_|^2^ is the surface tangential tensor, and *η* is a small value to ensure that the surface shear tractions are much smaller than the normal tractions. It is important to note that once the friction coefficient or shear force at Li‐SE interface is experimentally measured, the mechanical behavior of a frictional interface could be approximated by assigning a specific value to *η*.

It is also noted that at the Li‐SE interface, tetragonal‐like LLZO can form,^[^ ^33^ ^]^ inducing tensile stress on the surfaces of SE particles. According to numerical results from Malavé et al.,^[^ ^34^ ^]^ the maximum tensile stress generated by interphase formation can reach several hundred MPa when charging at a high current over a duration of 3600 s. However, based on this estimation, in the present study, the stress induced by interphase formation is only a few MPa during a short charging period of just a few seconds. This stress level is insufficient to influence crack formation and propagation. Therefore, in the current simulation, the effect of interphase formation on mechanical deformation is neglected.

### 2.3 Reaction Kinetics for Li Plating Considering the Stress Contribution

For the electrochemical reactions, we adopted the expression of Bazant^[^ ^35^ ^]^ to formulate the reaction rate, *r*, given by

(5) $$\begin{array}{cc} r & =k^{0}(a_{R}\exp\left(\frac{\left(1-\rho \right)\left(\mu_{R}^{\mathrm{ex}}-\mu_{P}^{\mathrm{ex}}\right)}{RT}\right)\\ & \;-a_{P}\exp\left(-\frac{\rho \left(\mu_{R}^{\mathrm{ex}}-\mu_{P}^{\mathrm{ex}}\right)}{RT}\right)) \end{array}$$

where *k*_0_ is the kinetics constant; *ρ* is the asymmetric parameter; *a*_R_ and *a*_P_ are the activity of reactants and products, respectively; $\mu_{R}^{\mathrm{ex}}-\mu_{P}^{\mathrm{ex}}$ is the difference of excess chemical potential between reactants and products. Considering the reaction Li^+^ + e^−^ → Li, $a_{R}=\exp\left(\mu_{\mathrm{chem}}^{Li^{+}}/RT\right)$ and $a_{P}=\exp\left(\mu_{\mathrm{chem}}^{\mathrm{Li}}/RT\right)$, where $\mu_{\mathrm{chem}}^{Li^{+}}$, $\mu_{\mathrm{chem}}^{\mathrm{Li}}$, are the chemical potential of Li ions in SE and atoms in Li deposit, respectively; *R* and *T* are the ideal gas constant and thermodynamic temperature, respectively. The difference in excess chemical potential between reactant and products, $\mu_{R}^{\mathrm{ex}}-\mu_{P}^{\mathrm{ex}}$, is expressed as

(6) $$\begin{array}{cc} \mu_{R}^{\mathrm{ex}}-\mu_{P}^{\mathrm{ex}} & =F\left(\varphi_{\mathrm{SE}}-\varphi_{\mathrm{CC}}\right)\\ & \;-\left(\psi^{\mathrm{mech}}-\det\left(F\right)\sigma \right){\left(F^{\mathrm{vp}}F^{\mathrm{vol}}\right)}^{-1}\frac{\partial \left(F^{\mathrm{vp}}F^{\mathrm{vol}}\right)}{\partial c_{2}^{\mathrm{Li}}} \end{array}$$

where *F* is Faraday's constant; *φ*_SE_ and *φ*_CC_ are the electric potential of SE and CC, respectively. Equation (6) introduces the effects of conformational volume change and deformation energy in the chemical potential. They are the consequence of large deformation. If the small deformation is assumed, the excess‐potential difference reduces to 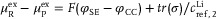, where *tr*(•) means the trace of a tensor. In this case, the effects of conformational volume change and deformation energy becomes insignificant.

### 2.4 Governing Equations

When the contact compressive stress is lower than the yield strength, the layer‐by‐layer deposition of Li atoms at the Li‐electrolyte interface displaces the existing Li layers, resulting in the growth of Li whiskers, also known as the root growth mode (as illustrated in Figure 1b). In this scenario, the driving force for Li whisker growth can be expressed in terms of the electroplating reaction rate, as defined by Chen et al.^[^ ^36^ ^]^ Since the elongation rate of Li whiskers corresponds to the rates of CC movement and gap widening, the driving terms governing the movement of the CC interface and the CC‐gap interface remain consistent with those describing Li whisker growth. Consequently, the governing equations for phase evolution can be expressed in the following form

(7) $$\left\{\begin{array}{cc} \frac{\partial p_{1}}{\partial t} & =-M\left(-\kappa \nabla^{2}p_{1}+m\left({\left(p_{1}\right)}^{3}-p_{1}+3p_{1}\sum_{j\neq 1}^{N}{\left(p_{j}\right)}^{2}\right)\right)\\ \frac{\partial p_{2}}{\partial t} & =-M\left(-\kappa \nabla^{2}p_{2}+m\left({\left(p_{2}\right)}^{3}-p_{2}+3p_{2}\sum_{j\neq 2}^{N}{\left(p_{j}\right)}^{2}\right)\right)\\ & \;+4h_{2}h_{3}\frac{r}{c_{\mathrm{ref}}^{\mathrm{Li}}}\\ \frac{\partial p_{3}}{\partial t} & =-M\left(-\kappa \nabla^{2}p_{3}+m\left({\left(p_{3}\right)}^{3}-p_{3}+3p_{3}\sum_{j\neq 3}^{N}{\left(p_{j}\right)}^{2}\right)\right)\\ & \;-4h_{3}\left(h_{2}+h_{4}\right)\frac{r}{c_{\mathrm{ref}}^{\mathrm{Li}}}\\ \frac{\partial p_{4}}{\partial t} & =-M\left(-\kappa \nabla^{2}p_{4}+m\left({\left(p_{4}\right)}^{3}-p_{4}+3p_{4}\sum_{j\neq 4}^{N}{\left(p_{j}\right)}^{2}\right)\right)\\ & \;+4h_{3}h_{4}\frac{r}{c_{\mathrm{ref}}^{\mathrm{Li}}}\left(4h_{2}h_{3}{\bar{\sigma }}_{\mathrm{mises}}\leq \sigma_{y}^{\mathrm{Li}}\right) \end{array}\right.$$

where $4h_{2}h_{3}r/c_{\mathrm{ref}}^{\mathrm{Li}}$ is the driving force for the evolution of Li, which is mediated by reaction kinetics; $4h_{3}\left(h_{2}+h_{4}\right)r/c_{\mathrm{ref}}^{\mathrm{Li}}$ is the rigid body movement of flexible CC; $4h_{3}h_{4}r/c_{\mathrm{ref}}^{\mathrm{Li}}$ decribes the widening of gap between SE and CC; $4h_{2}h_{3}{\bar{\sigma }}_{\mathrm{mises}}$ denotes the average contact Mises stress along Li‐CC interface; $4h_{2}h_{3}{\bar{\sigma }}_{\mathrm{mises}}\leq \sigma_{y}^{\mathrm{Li}}$ indicates that the root‐growth only occurs when the average contact mises stress is smaller than the yield strength of Li. Since the deposited Li atoms do not interstitially insert into host lattice, it leads $\mu_{R}^{\mathrm{ex}}-\mu_{P}^{\mathrm{ex}}$ to be $\mu_{R}^{\mathrm{ex}}-\mu_{P}^{\mathrm{ex}}=F\left(\varphi_{\mathrm{SE}}-\varphi_{\mathrm{CC}}\right)+tr\left(4h_{1}h_{2}\sigma \right)/c_{\mathrm{ref},\;2}^{\mathrm{Li}}$, where 4*h*_1_ *h*_2_ **σ** is the contact stress tensor between the SE and lithuim.

According to the mass conservation laws for Li ions in SE, it leads to the reaction diffusion equation of Li ions, as follows

(8) $$\begin{array}{cc} & \frac{\partial \mu_{\mathrm{chem}}^{Li^{+}}}{\partial t}={\left(\sum_{\alpha =1}^{N}h_{\alpha }\frac{\partial c_{\alpha }^{Li^{+}}}{\partial \mu_{\mathrm{chem}}^{Li^{+}}}\right)}^{-1}\\ & \;\times \left(\begin{array}{c} \nabla \left(D_{Li^{+}}^{\mathrm{diff}}{\left(\sum_{\alpha =1}^{N}h_{\alpha }\frac{\partial c_{\alpha }^{Li^{+}}}{\partial \mu_{\mathrm{chem}}^{Li^{+}}}\right)}^{-1}\left(\nabla \mu_{\mathrm{chem}}^{Li^{+}}-F\nabla \varphi_{\mathrm{SE}}\right)\right)\\ -r-\sum_{\alpha =1}^{N}\frac{\partial h_{\alpha }}{\partial t}c_{\alpha }^{Li^{+}} \end{array}\right) \end{array}$$

where $D_{Li^{+}}^{\mathrm{diff}}$ is the diffusion matrix of Li ion; $c_{\alpha }^{*}$ is the phase concentration of component ⁎ in *α* phase, which is a fictitious concentration, having the following relationship with the actual concentration of component, *c* ^⁎^, as $c^{*}=\sum_{\alpha =1}^{N}h_{\alpha }c_{\alpha }^{*}$. Considering the fast diffusion‐pathways along interface and surface, $D_{Li^{+}}^{\mathrm{diff}}$, is expressed as: 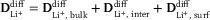, where $D_{Li^{+},\;\mathrm{bulk}}^{\mathrm{diff}}$, $D_{Li^{+},\;\mathrm{inter}}^{\mathrm{diff}}$, and $D_{Li^{+},\;\mathrm{surf}}^{\mathrm{diff}}$ are, respectively, the diffusive matrix of Li ion in the body of SE, along the Li‐SE and CC‐SE interfaces, and along the surface of SE.

The Poisson's equation is employed to govern the electropotential field, *φ*, given by

(9) $$\nabla \left(\varepsilon \nabla \varphi \right)=0$$

where *ε* = *ε*_S_(*h*_1_(1−*χ*(*θ*))+*h*_3_) + *ε*_SE_ *h*_2_ + *η*(*h*_1_ *χ*(*θ*)+*h*_4_) is the effective electric conductivity of the medium with *ε*_S_ and *ε*_SE_ being the conductivities of the conductor and SE; *η* is to ensure that the unfilled gap and crack are electrically insulated.

If the Li deposition is under a strong mechanical constraint, as shown in Figure 1c, leading to viscoplastic flow and SE cracking, the governing equations need to have additional terms to describe the material flow. Hence, Equations (7) are modified as

(10) $$\begin{array}{c} \left\{\begin{array}{cc} \frac{\partial p_{i}}{\partial t} & =-M(-\kappa \nabla^{2}p_{i}+m\left({\left(p_{i}\right)}^{3}-p_{i}+3p_{i}\sum_{j\neq i}^{N}{\left(p_{j}\right)}^{2}\right)\\ & \;+\sum_{\alpha =1}^{N}\frac{\partial h_{\alpha }}{\partial p_{i}}\left(\psi_{\alpha }^{\mathrm{chem}}-\mu^{\mathrm{Li}}c_{\alpha }^{\mathrm{Li}}-\mu^{Li^{+}}c_{\alpha }^{Li^{+}}\right))\\ & \;\times \left(4h_{2}\left(1-h_{2}\right){\bar{\sigma }}_{\mathrm{mises}}>\sigma_{y}^{\mathrm{Li}}\right) \end{array}\right. \end{array}$$

where $\psi_{\alpha }^{\mathrm{chem}}-\mu^{\mathrm{Li}}c_{\alpha }^{\mathrm{Li}}-\mu^{Li^{+}}c_{\alpha }^{Li^{+}}$ is the additional driving force for the evolution of order parameter filed; $4h_{2}\left(1-h_{2}\right){\bar{\sigma }}_{\mathrm{mises}}$ represents the average contact stress between the Li and other phase; $4h_{2}\left(1-h_{2}\right){\bar{\sigma }}_{\mathrm{mises}}>\sigma_{y}^{\mathrm{Li}}$ indicates that viscoplastic flow only occurs when the average contact stress is larger than the yield strength of Li.

Since the variation of driving force is owing to the diffusion and reaction of Li, it leads to the reaction diffusion equation of Li atoms as follows

(11) $$\begin{array}{cc} & \frac{\partial \mu_{\mathrm{chem}}^{\mathrm{Li}}}{\partial t}={\left(\sum_{\alpha =1}^{N}h_{\alpha }\frac{\partial c_{\alpha }^{\mathrm{Li}}}{\partial \mu_{\mathrm{chem}}^{\mathrm{Li}}}\right)}^{-1}\\ & \;\times \left(\begin{array}{c} \nabla \left(D_{\mathrm{Li}}^{\mathrm{diff}}{\left(\sum_{\alpha =1}^{N}h_{\alpha }\frac{\partial c_{\alpha }^{\mathrm{Li}}}{\partial \mu^{\mathrm{Li}}}\right)}^{-1}\left(\begin{array}{c} \nabla \mu_{\mathrm{chem}}^{\mathrm{Li}}-\\ \nabla \left(\begin{array}{c} \left(\psi^{\mathrm{mech}}-\det\left(F\right)\sigma \right)\\ {\left(F^{\mathrm{vp}}F^{\mathrm{vol}}\right)}^{-1}\frac{\partial \left(F^{\mathrm{vp}}F^{\mathrm{vol}}\right)}{\partial c_{2}^{\mathrm{Li}}} \end{array}\right) \end{array}\right)\right)\\ +r-\sum_{\alpha =1}^{N}\frac{\partial h_{\alpha }}{\partial t}c_{\alpha }^{\mathrm{Li}} \end{array}\right) \end{array}$$

In Equation (11), $D_{\mathrm{Li}}^{\mathrm{diff}}$ is the diffusion matrix of Li atom. It is expressed as: $D_{\mathrm{Li}}^{\mathrm{diff}}=D_{\mathrm{Li},\;\mathrm{bulk}}^{\mathrm{diff}}+D_{\mathrm{Li},\;\mathrm{inter}}^{\mathrm{diff}}+D_{\mathrm{Li},\;\mathrm{surf}}^{\mathrm{diff}}$, where $D_{\mathrm{Li},\;\mathrm{bulk}}^{\mathrm{diff}}$, $D_{\mathrm{Li},\;\mathrm{inter}}^{\mathrm{diff}}$, and $D_{\mathrm{Li},\;\mathrm{surf}}^{\mathrm{diff}}$ are, respectively, the diffusivity matrix of Li atom in the host lattice, along the Li‐SE and Li‐CC interfaces, and along the surface of Li. Equation (11) indicates that Li atom diffusion is driven by gradients in chemical potential and by stresses induced by Li insertion into the host lattice. Consequently, as Li atoms diffuse and reduce the fraction of inserted atoms, stress relaxation occurs, leading to the flow of Li to fill gaps, cavities, and cracks. This behavior aligns with the fundamental mechanism of creep.

Based on the fracture PF theory, the governing equation of cracking in SE can be expressed as^[^ ^37^ ^]^

(12) $$\frac{G_{f}}{l}\left(\theta -l^{2}\nabla^{2}\theta \right)=2\left(1-\theta \right)\;Yh_{1}$$

where *G*_f_ is the energy dissipated upon the creation of a unit on the fracture surface; *l* denotes the thickness of the Crack‐SE interface, and it can be regarded as a pure numerical parameter; *Y* is the effective crack driving force; and *h*_1_ ensures the cracking only occur in SE.

**Figure 2:**
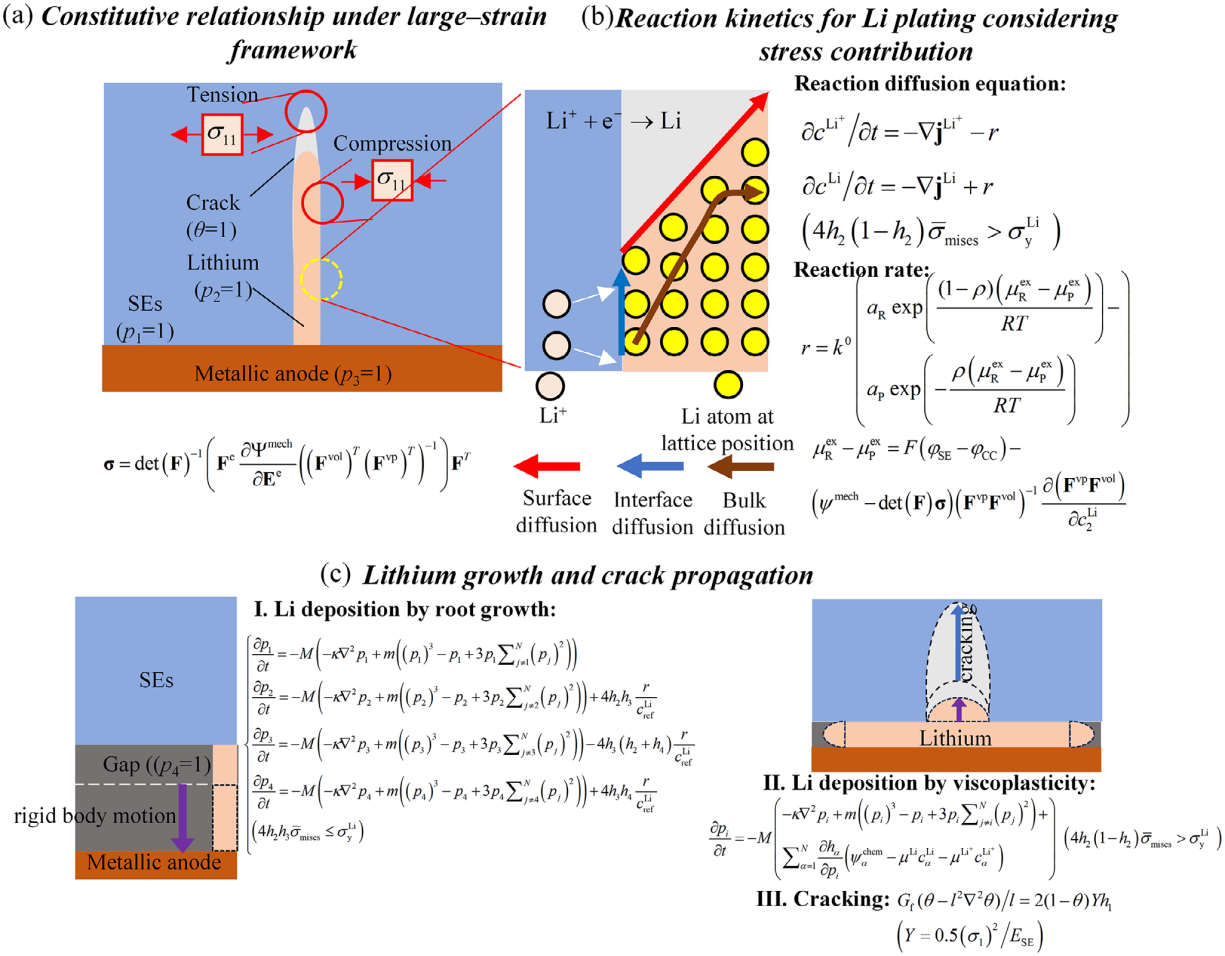
Schematic diagram of the theoretical model. a) Constitutive relationship under the large‐strain framework: This framework accounts for both chemical strain and viscoplastic deformation. Chemical strain arises from the electroplating reaction, where Li atoms are deposited in quantities exceeding the stoichiometric amount of Li metal within a confined space. Consequently, the constitutive relationship describes the deformation behavior associated with electroplating, wherein excess Li atoms are interstitially inserted into the host lattice, leading to compression of defect sidewalls. b) Plating reaction kinetics: This subfigure illustrates concentration changes driven by the migration of Li ions and atoms, as well as deposition reactions. Variations in Li atom concentration induce chemical strain. c) Phase‐field evolution equation for the electroplating process: Li deposition follows the root growth mode when the contact compressive stress is below the yield strength, as described by equation (I) in (c). When the contact compressive stress reaches the yield strength, Li deposition transitions to the viscoplastic flow mode, as shown in Equation (II) in (c). Additionally, stress accumulation can lead to defect cracking, which is governed by the fracture PF equation, as illustrated in Equation (III) in (c).

Thus, by integrating the constitutive relationship (Equation (1)), the diffusion‐reaction equations for Li ions and atoms (Equations (8) and (11)), Poisson's equation (Equation (9)), the governing equations for the order parameter field (Equations (7) and (10)), and the fracture equation (Equation (12)), the processes of Li deposition and SE cracking are fully characterized. As illustrated in **Figure** **2**, we also provide a schematic diagram summarizing the theoretical modeling process.

## 3 Results and Discussion

The simulations were conducted using COMSOL Multiphysics software. The geometric and boundary conditions and the material parameters utilized in the simulations are detailed in the Supporting Information.

### 3.1 Low Electric‐Potential Plating with Variable Constraints

In the experiments conducted by Gao et al.,^[^ ^26^ ^]^ single‐crystal LLZO particles were used as SE to investigate electrodeposition behavior by constructing Li‐LLZO‐CC nanocells. Transmission electron microscopy (TEM) was employed to observe interface dynamics in cross‐section. To impose mechanical constraints during low electric‐potential plating, tungsten (W) was used as a probe. In situ TEM force measurements were performed using a TEM atomic force microscope (AFM) mount, with the W probe serving as the force application element. The AFM cantilever was affixed to the stationary side of the TEM stand, while the probe tip, connected to a piezoelectric controller, was pushed upward against the cantilever tip. During plating, the W tip bent under the compressive force exerted by the AFM cantilever tip. The applied force was determined using Hooke's law: *F* = *k*_s_∆*l*, where *k*_s_ is the spring constant of the probe cantilever (*k*_s_ = 5 N m^−1^ in the experiment^[^ ^26^ ^]^) and ∆*l* represents the displacement of the cantilever tip. Consequently, in our simulations, the mechanical constraint imposed by the W probe is idealized as an elastic constraint.

**Figure 3:**
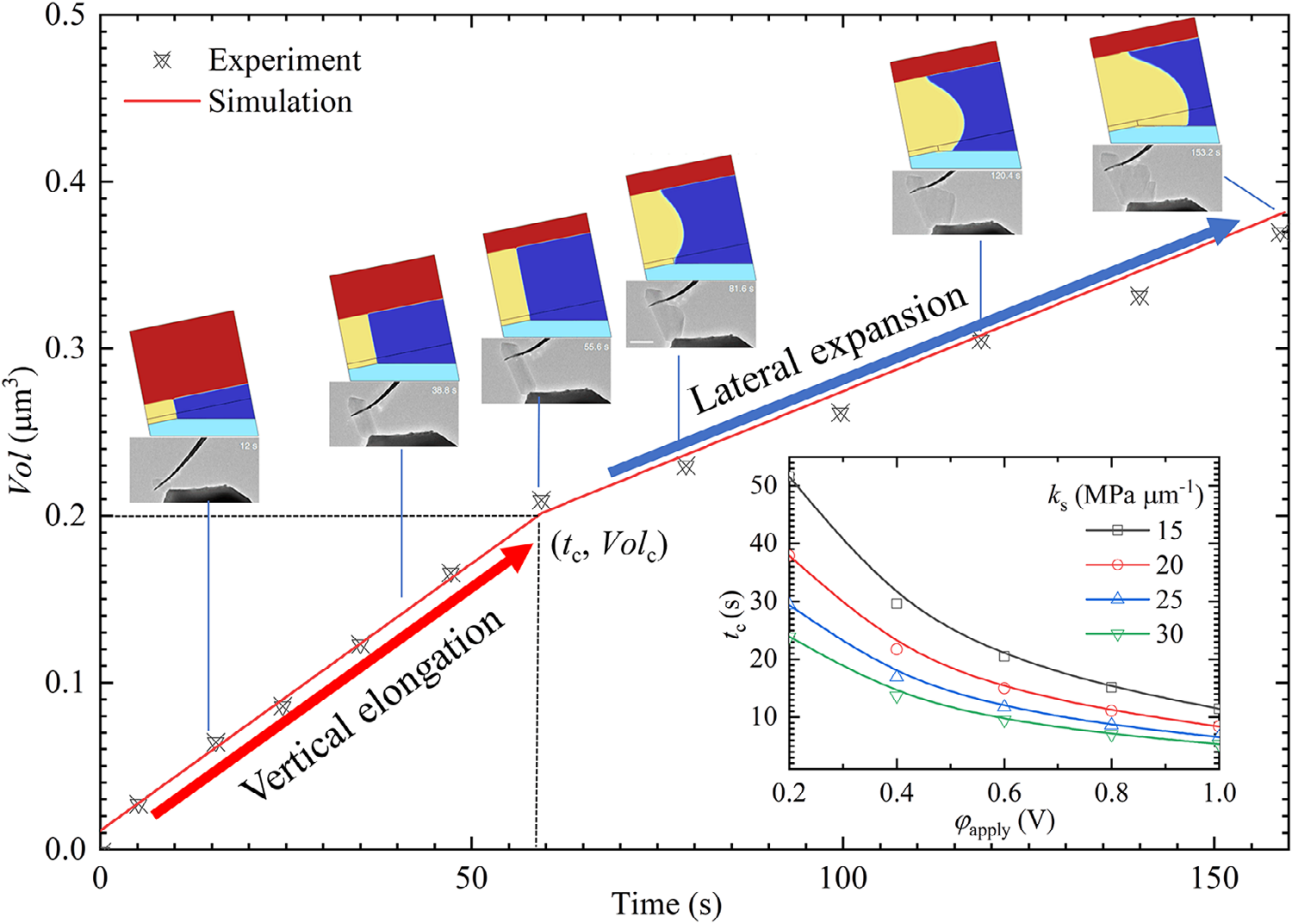
Entire process of morphology and volume evolution of deposited Li in low electric‐potential plating with variable constraint; the inset of Figure 3 quantitatively plots the critical time for root‐growth to shank‐expansion, *t*_c_, versus bias electric‐potential, *φ*_apply_, and spring constant, *k*_s_.

To implement the elastic constraint, a stack stress corresponding to the elongation of the Li whisker—equivalent to the displacement of the cantilever tip, ∆*l*—was applied to the upper surface of the CC. This stress was expressed as *σ*_sp_ = *k*_s_∆*l*. For detailed information regarding the simulation geometry, boundary conditions, and finite element mesh, please refer to Figure S1 in the Supporting Information. To validate the model's accuracy, the simulation results were compared with experimental data from Gao et al.,^[^ ^26^ ^]^ as presented in **Figure** **3**.

According to Equation (7), Li grows via layer‐by‐layer deposition, where each newly deposited layer displaces the previously deposited layers at the Li‐SE interface. This growth mode is referred to as root growth. As depicted in Figure 3, root growth results in the radial elongation of Li whiskers perpendicular to the SE surface, pushing the CC away from 0 to 58 s. Based on the established theory (Equation (7)), the deposition reaction governs root growth, and its rate is solely dependent on the reaction rate. Furthermore, according to the mechano‐chemical coupling theory (Equations (5) and (6)), stack pressure shifts the deposition overpotential to the left, thereby slowing the reaction rate. Consequently, as the CC is displaced, the stack pressure increases, gradually reducing the whisker elongation rate. However, both simulation and experimental results indicate that the elongation rate of Li whiskers remains unchanged despite increasing stack pressure. This observation contrasts with the findings of Zhang et al.,^[^ ^29^ ^]^ which reported a slowdown in whisker elongation with increasing stack pressure. This discrepancy may arise from differences in the electrochemical properties at the Li‐SE interfaces in the two experiments. The current study suggests that the overpotential at the Li‐SE interface was sufficiently large, rendering changes in overpotential due to increased stack pressure negligible. In contrast, Zhang et al.^[^ ^29^ ^]^ observed a low overpotential at the electroplating interface, where reductions in overpotential caused by increased stacking stress could not be ignored. Therefore, although the two observed phenomena differ, they are not inherently contradictory.

A transition in Li growth mode is observed as deposition time extends, shifting from whisker elongation to whisker expansion. This change results from variations in mechanical constraints and the plastic yielding of Li. Under low mechanical constraint conditions (when the contact stress is below the yield strength of Li), Li is deposited via root growth (Equation (7)). In contrast, under high mechanical constraints (when the contact stress reaches the yield strength of Li), newly deposited Li can no longer displace atomic layers at the interface and is instead deposited onto the free surface through atomic diffusion, a process known as dislocation climbing (Equations (10) and (11)). This growth mode is referred to as viscoplastic flow growth. At the moment of growth mode transition, the stack stress equals the yield strength of Li. Numerical calculations show a stack stress of 50 MPa, which is within the experimentally measured yield strength range of Li whiskers.^[^ ^29^ ^]^ Moreover, because viscoplastic flow is limited by atomic diffusion, whereas root growth is controlled by the reaction rate, the volumetric deposition rate of Li differs between the two growth modes. Both simulation and experimental results show that the volume change rate of Li is approximately three times higher in root growth mode compared to viscoplastic flow mode.

Another critical observation is that when Li is deposited via viscoplastic flow, it does not preferentially deposit at the triple‐phase point (TPP) of the Li‐SE‐gap. Instead, it is primarily deposited onto the free surface of the whiskers, as illustrated in Figure 3 (58–81 s). Li is only preferentially deposited at the TPP when the free surface curvature becomes significant. This occurs because the surface energy at the TPP is initially greater than that of the flat free surface, making TPP deposition unfavorable. However, as Li deposition raises the free surface, its surface energy eventually exceeds that of the TPP, causing Li to be preferentially deposited at the TPP, as observed after 81 s in Figure 3. This simulation result is consistent with experimental observations.^[^ ^26^ ^]^ Additionally, we simulated the stress evolution during deposition, as illustrated in Figure S2 in the Supporting Information.

### 3.2 High Electric‐Potential Plating with Ridge Constraints

Copper (Cu) was used as a probe in high electric‐potential plating experiments,^[^ ^26^ ^]^ exhibiting no deformation during in situ tests. Therefore, in the simulation, the mechanical constraint imposed by the Cu probe was idealized as a rigid constraint. Additionally, the simulations assume the presence of a nanoscale defect on the SE surface, prefilled with Li. The defect tip is modeled as a circular shape, with its diameter representing its width. In the simulation, the defect width is kept constant at 5 nm, while its depth varies.

#### 3.2.1 SE Particle Tightly Constrained by Other Particles

**Figure 4:**
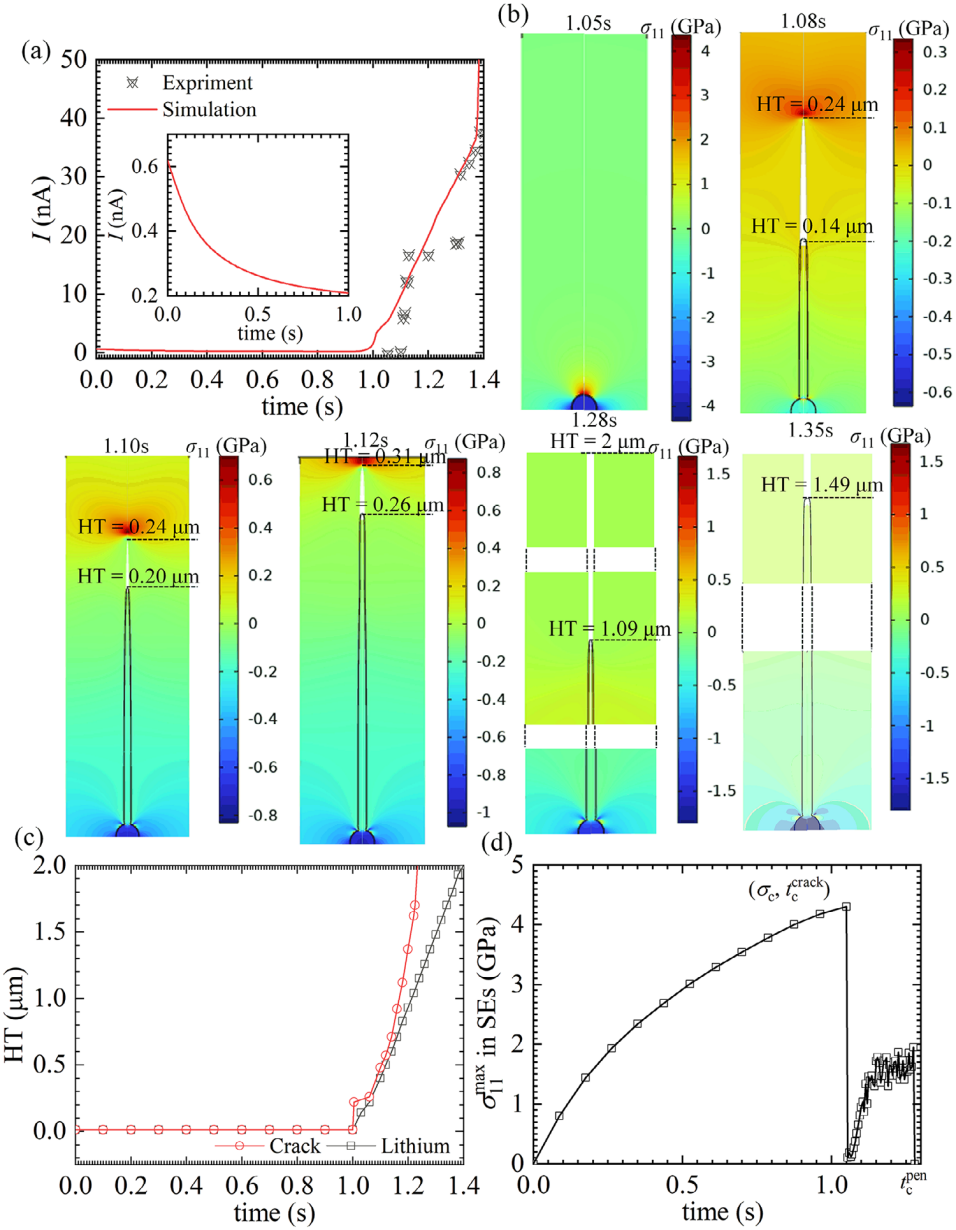
a) Evolution of current. b) Plating induced cracking in high electric‐potential with ridge constraints for the particles tightly constrained by other particles. c) Height of crack and Li dendrite with time. d) Evolution of tensile stress, *σ*_11_, at the tip of defect‐crack system. And the inset of (a) depicts the evolution of current before cracking in details.

For the SE particle tightly constrained by surrounding particles, detailed information regarding the geometry, boundary conditions, and finite element mesh is provided in Figure S3 of the Supporting Information. As shown in **Figure** **4**a, we quantitatively compared the simulated and experimentally measured deposition current densities^[^ ^26^ ^]^ to validate the accuracy of the model.

According to the developed model, the crack initiation aligns with the pressurized crack model description. The localized fracture strength is related to defect size

(13) $$\sigma_{c}\geq \frac{K_{\mathrm{IC}}}{\varpi \sqrt{\pi d_{s,\mathrm{ini}}}}$$

where *σ*_c_ is the localized fracture strength, *K*_IC_ is fracture toughness of a Mode‐I crack, *d*_s,ini_ is the initial length of crack, and *ϖ* is the geometric factor.

As shown in Figure 4b, the tensile stress required to induce cracking in nanoscale defects (≈20 nm) can reach the GPa order of magnitude at 1.05 s. This result aligns with the theoretical prediction by Gao et al.^[^ ^26^ ^]^ Simultaneously, the plating stress (compressive stress inside the defects) also reaches the GPa scale, leading to a significant shift in the deposition overpotential. Consequently, before cracking occurs, the current density decreases substantially with increasing deposition time, as illustrated in the inset of Figure 4a.

Plating stresses on the order of GPa indicate that a significant amount of elastic energy is stored within the defects. Once the tensile stress reaches the localized fracture strength, this elastic energy is rapidly released, triggering crack initiation. To accurately capture this process, the time step in the simulation is set to 10^−6^ s during the first 0.001 s of crack initiation. As shown in Figure 4c, the crack length instantaneously increases from 0 to 0.24 µm at 1.05 s, whereas the length of the Li dendrite remains nearly unchanged during the same time period. This indicates that crack propagation and Li dendrite growth are not synchronized.

After the crack initiates, Li flows into the crack, leading to a rapid release of elastic energy. This energy release reduces the tensile stress at the crack tip, as shown in Figure 4d for 1.05 s, thereby preventing further crack propagation. In Figure 4b, the Li dendrite elongates by 0.2 µm between 1.05 and 1.10 s, while the crack length remains unchanged. Subsequently, as more Li is deposited at the root, the tensile stress at the crack tip gradually increases. Once the tensile stress reaches the macroscopic fracture strength, the crack begins to propagate again. This type of cracking, known as the wedge‐opening mode, accelerates crack growth and leads to the rapid penetration of SE particles within 0.3 s, as shown in Figure 4c.

This behavior differs from the simulations by Monismith et al.,^[^ ^25^ ^]^ in which the crack propagates at a constant velocity. Although it was not possible to trace the crack propagation process in situ during experiments, the theory proposed by Ning et al.^[^ ^15^, ^16^ ^]^ suggests that crack initiation and propagation occur through pressurized cracking and wedge opening, respectively, resulting in a nonconstant crack propagation velocity.

As shown in Figure 4c, the interval between these two stages is less than 0.05 s, which is consistent with the findings of Gao et al.^[^ ^26^ ^]^ This also indicates that crack propagation and Li dendrite penetration occur almost simultaneously. This synchronization arises because when SE particles fracture, they are unable to widen the crack through rigid body displacement, thus limiting the available space for Li deposition. As a result, Li is deposited only within the narrow crack, leading to its rapid eruption and penetration into the solid electrolyte. This phenomenon also explains the sudden increase in deposition current density following cracking, as observed in Figure 4a.

#### 3.2.2 Effect of Mechanical Properties of SE to Plating‐Induced Cracking and Short‐Circuiting

The formation of Li dendrites occurs through crack initiation, which is influenced by defect size and operating conditions (e.g., bias electric potential). Therefore, this study further investigates the effects of defect size and bias electric potential on crack initiation. The geometrical dimensions and boundary conditions used in the simulations are consistent with those in Section 3.2.1.

**Figure 5:**
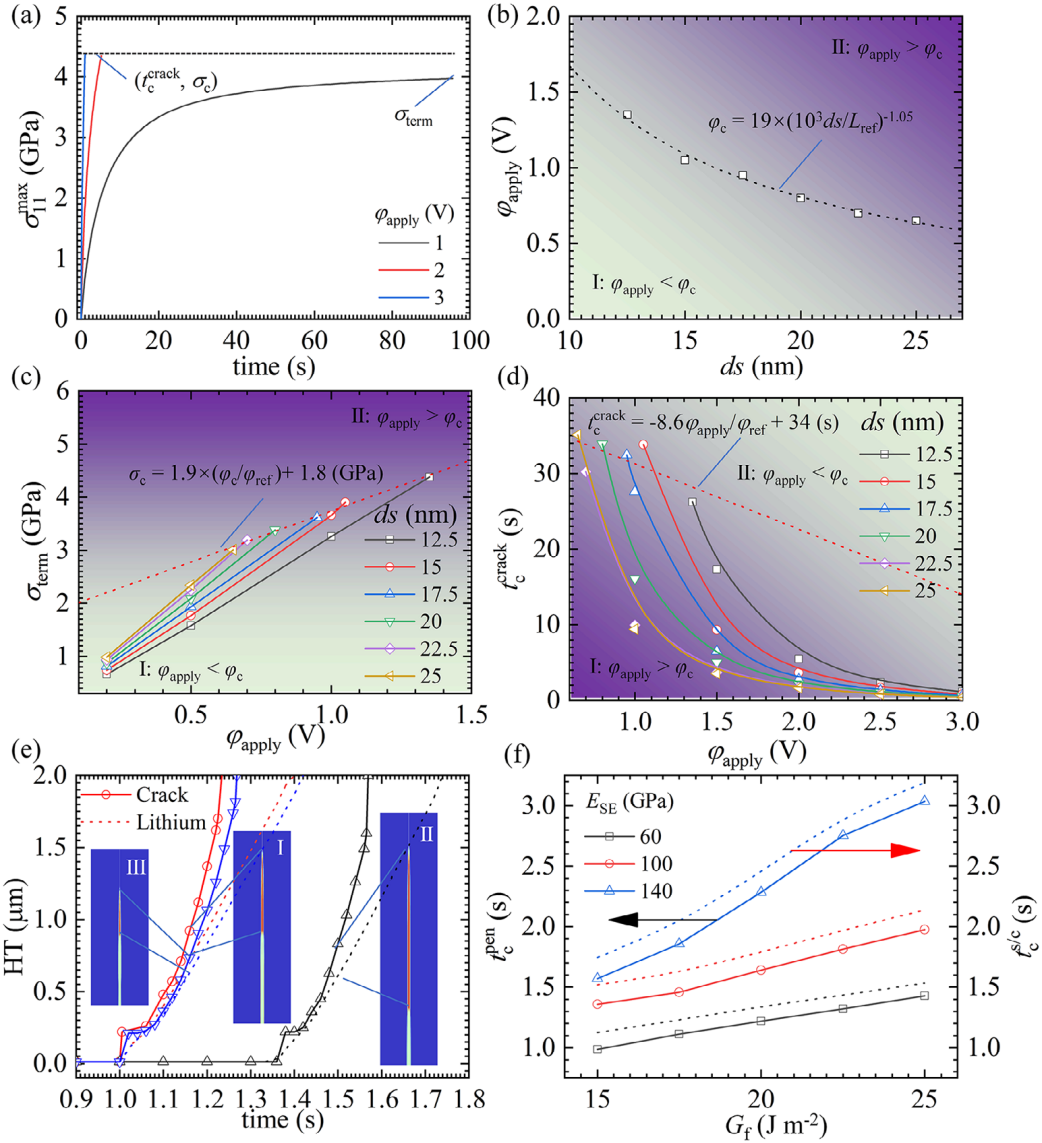
a) Evolution of tensile stress at the defect tip under different bias electric potentials before cracking. b) Critical bias electric potential as a function of defect size. c) Tensile stress at the defect tip as a function of defect size and applied bias electric potential at the termination of the reaction. d) Critical fracture time as a function of defect size and applied bias electric potential. e) Process of plating‐induced cracking for three different cases. f) Relationship between the time of crack penetration and the time of short‐circuiting as a function of the stiffness and fracture energy density of the SE, respectively.

As shown in **Figure** **5**a, the maximum tensile stress near the defect increases with both bias electric potential and plating time. At high bias electric potentials (2 and 3 V), the tensile stress eventually reaches the local fracture strength, leading to crack initiation. However, at a lower bias electric potential (1 V), the tensile stress reaches a saturation value below the local fracture strength, preventing crack initiation. This stress evolution is attributed to mechano‐chemical coupling and suggests the existence of a critical bias electric potential. When the bias electric potential is below this critical value, the deposition process does not induce crack initiation.

A quantitative summary of the relationship between defect size and critical bias electric potential is provided in Figure 5b. Additionally, the relationships between critical bias electric potential and local fracture strength, as well as between critical bias electric potential and critical fracture time, are presented in Figure 5c,d, respectively. These findings provide theoretical guidance for optimizing pore size and operating conditions.

The growth of Li dendrites occurs through crack propagation, which is influenced by the stiffness and toughness (fracture energy density) of the SE. Therefore, this study also examines the effects of stiffness and fracture energy density on crack and dendrite propagation. The simulations adopt geometrical configurations and boundary conditions consistent with those in Section 3.2.1.

Figure 5e illustrates the penetration of cracks and Li dendrites through the SE under three different conditions:

**Scenario 1**: SE stiffness of 120 GPa and fracture energy density of 15 J m^−2^.
**Scenario 2**: SE stiffness of 140 GPa and fracture energy density of 15 J m^−2^.
**Scenario 3**: SE stiffness of 120 GPa and fracture energy density of 25 J m^−2^.

The results indicate that increasing the stiffness of the SE suppresses crack initiation but promotes crack propagation, while increasing the fracture energy density inhibits crack propagation. However, neither increasing stiffness nor toughness alone can fully prevent the rapid propagation of cracks and Li dendrites. As shown in Figure 5f, when the stiffness and fracture energy density were increased from 60 GPa and 15 J m^−2^ to 140 GPa and 25 J m^−2^, respectively, the time required for cracks and Li dendrites to penetrate the SE increased only marginally, from 1.0 and 1.1 s to 3.0 and 3.2 s, respectively.

#### 3.2.3 CC in Contact with Isolated SE Particles

**Figure 6:**
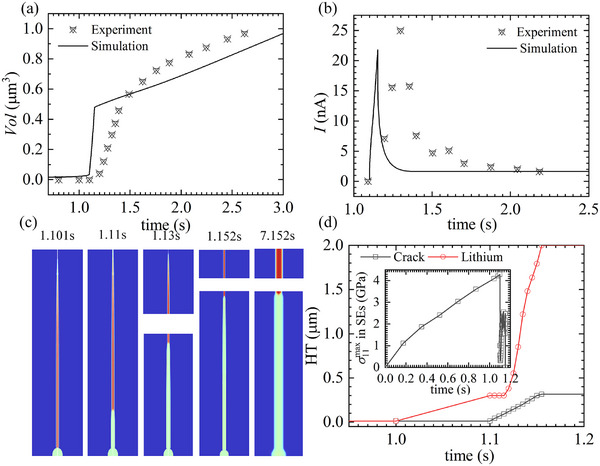
Evolution curves illustrating: a) The deposited Li volume over time. b) The deposition current density over time. c) The process of Li dendrite growth and SE cracking; and d) the evolution of Li dendrite and crack length over time. The inset in (d) presents the evolution curve of the maximum tensile stress at the crack tip over time.

Our research also investigated the propagation of cracks and Li dendrites within individual SE particles. The details of the simulation setup, including geometries, boundary conditions, and finite element meshes, are provided in Figure S6 of the Supporting Information. To validate the accuracy of our findings, we compared the simulation results with experimental measurements,^[^ ^26^ ^]^ as shown in **Figure** **6**a,b.

As depicted in Figure 6c, the process of plating‐induced crack formation, from crack initiation (1.1 s) to the penetration of SE particles (1.15 s), closely resembles the scenario discussed in Section 3.2.1. However, the simulation results exhibit significant differences after the crack fully penetrates the SE particles (time greater than 1.15 s). At this stage, the growth mode of Li dendrites transitions from elongation along the axial direction of the crack to radial expansion perpendicular to the crack's sidewalls. Simultaneously, the crack width increases. This change is attributed to the removal of mechanical constraints on the deposited Li by the crack sidewalls when the SE particles split into two halves. According to the developed model, Li dendrite growth shifts from the viscous flow growth mode to the root growth mode in this scenario, where Li is directly deposited on the crack's sidewalls while pushing the SE particles apart. Consequently, as shown in Figure 6d, the Li dendrite length ceases to increase after the crack penetrates the SE particles. This observation is consistent with the findings of Gao et al.,^[^ ^26^ ^]^ where significant crack widening was observed in experiments with isolated SE particles, preventing short‐circuiting. Conversely, in SE particles surrounded by other particles, only a “hidden” narrow crack was observed, and the crack penetration and Li dendrite growth into the SE occurred almost simultaneously.

A comparison of deposition current densities reveals that when SE particles are constrained by surrounding particles, the deposition current density increases steadily until short‐circuiting occurs after cracking. In contrast, for isolated SE particles, the deposition current exhibits a pulsed pattern. This indicates that in particle‐constrained SE, Li continuously fills cracks with a high‐velocity eruption until it fully penetrates the SE. Conversely, in isolated SE particles, Li deposition initially involves a brief eruption before transitioning to the root growth mode.

Comparing the results of the two simulations leads to a counterintuitive conclusion: to extend the lifespan of LMSSBs, minimizing the mechanical constraints on the SE is more effective than reducing defect size or increasing the hardness and toughness of the SE. This approach is viable as long as good contact is maintained between the electrode and the SE interface. The reason is that reducing mechanical constraints enables a transition from viscoplastic flow deposition to the root growth mode, thereby preventing the rapid eruption of Li within cracks.

Following this principle, several strategies can be proposed to enhance the lifespan of LMSSBs. For instance, a composite structure composed of a polymer lattice with internally filled SE particles could be used instead of a single‐material SE.^[^ ^38^ ^]^ In this configuration, crack propagation is inhibited by the polymer lattice, reducing the likelihood of Li penetration through the SE. Simultaneously, the flexible polymer lattice decreases the mechanical constraints imposed on Li dendrites, facilitating crack widening, and creating additional space for Li deposition, ultimately reducing short‐circuiting caused by Li eruption.

### 3.3 High Electric‐Potential Plating without Constraints

Another approach to reducing mechanical constraints is the use of carbon nanotubes (CNTs) as the CC. In this configuration, the interior of the CNT serves as a space for Li deposition, effectively mitigating mechanical constraints. To investigate Li deposition behavior in CNT‐based systems, we conducted numerical simulations and compared the results with experimental data from Gao et al.^[^ ^26^ ^]^ The simulation geometry, boundary conditions, and finite element mesh are detailed in Figure S9 of the Supporting Information.

**Figure 7:**
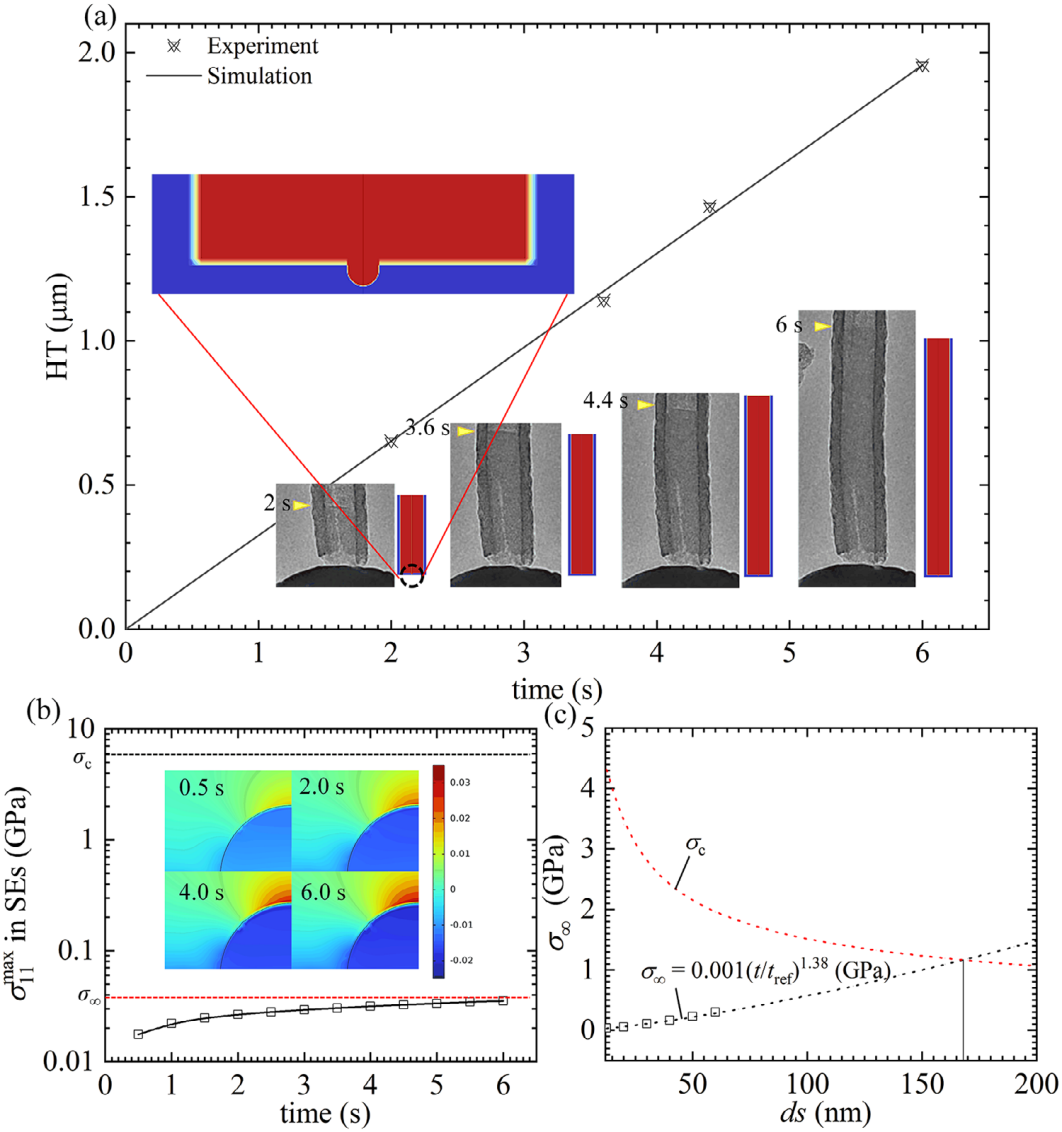
a) Growth of Li within CNTs. b) Stress evolution at the defect tip during plating. c) Evolution of stress at the defect tip after long‐term plating and comparison with the localized fracture strength of SE as a function of defect size.

As illustrated in **Figure** **7**a, the simulation results for the height of Li deposited within the CNT defect over time at high bias electric potential closely match the experimental measurements. Due to the high friction at the CNT wall, newly deposited Li atoms on the SE surface cannot displace the existing atomic layer. Consequently, Li is deposited within the CNT via a viscoplastic flow mode. If a defect on the SE surface is connected to the CNT, plating stress develops inside the defect. However, this stress can be effectively relieved, as the interior of the CNT provides additional space for the viscoplastic flow of Li. Figure 7b presents the evolution of tensile stress at the defect tip over deposition time. The results indicate that the tensile stress approaches a saturation value that remains below the localized fracture strength, suggesting that CNTs effectively prevent SE fracture in this scenario. Furthermore, we quantitatively predicted the relationship between the saturation stress and the initial defect size and compared it with the local fracture strength (see Figure 7c). The simulation results suggest that SE fracture can be avoided at high bias electric potential if the defect size is smaller than 165 nm. This finding provides a theoretical basis for the design of LMSSBs.

## 4 Conclusion

This paper introduces a unified mechano‐chemical PF model to provide insights into Li deposition and electrolyte cracking during the charging process of LMSSBs. Unlike existing models,^[^ ^18^, ^19^, ^20^, ^21^, ^22^, ^23^, ^24^, ^25^ ^]^ this model accounts for the influence of mechanical constraints on Li deposition modes and the contact between Li and defect sidewalls. Crack propagation is described in two stages—initiation and extension—using the pressurized crack model and the wedge opening model, respectively. Additionally, the model incorporates a coupled mechano‐chemical theory within a large deformation framework. To validate its accuracy, the simulation results have been quantitatively compared with experimental data under various conditions.

The study investigates Li deposition under low electric potential with elastic mechanical constraints. The findings reveal a transition in Li whisker growth from root growth mode to viscoplastic flow mode. Specifically, when the contact stress is below the yield strength of Li, deposition occurs via root growth, resulting in whisker elongation. Conversely, when the contact stress equals the yield strength of Li, deposition proceeds via viscoplastic flow, leading to whisker expansion.

Furthermore, Li deposition under high electric potential with rigid mechanical constraints is examined. When SE particles are surrounded by other particles, Li erupts at high velocity through narrow cracks via viscoplastic flow, leading to short‐circuit failure. However, in isolated SE particles, Li deposition transitions to root growth mode after crack penetration, preventing dendrite‐induced failure.

The study also quantitatively evaluates the effects of defect size, bias electric potential, particle stiffness, and toughness on crack initiation, propagation, and Li growth. While it is widely acknowledged that reducing defects can help mitigate SE cracking, the simulation results indicate that for defects of a specific size, there exists a critical bias electric potential threshold. When this threshold is exceeded, SE cracking occurs. Additionally, SE stiffness and toughness influence crack propagation. However, the findings suggest that increasing SE stiffness and toughness alone is ineffective in preventing the rapid expansion of cracks and Li dendrites.

Based on these findings, the study reaches a counterintuitive conclusion: the most effective strategy to extend the lifespan of LMSSBs is to reduce mechanical constraints on the SE rather than decreasing defect size or increasing SE hardness and toughness. This approach is viable as long as good contact is maintained between the electrode and the SE interface. By minimizing mechanical constraints, Li deposition shifts from viscoplastic flow mode to root growth mode, thereby preventing the rapid eruption of Li within cracks.

## Author Contributions

C.L. and H.H.R. conceived the idea. C.L. was responsible for model establishment. C.L. conducted simulations. C.L., H.W.G., H.H.R., and M.S.W. analyzed the results and wrote the manuscript.

## Conflict of Interest

The authors declare no competing interests.

## Supporting information



Supporting Information

## Data Availability

The data that support the findings of this study are available from the corresponding author upon reasonable request.
