# Supplementary material for: Comprehensive Study of Li Deposition and Solid Electrolyte Cracking by Integrating Simulation and Experimental Data
Source: Adv Sci (Weinh). 2025 Mar 16;12(25):2501434. doi: 10.1002/advs.202501434 (PMC12225003; doi:10.1002/advs.202501434)
Supplement: Supplementary file 1 — Supporting Information [file ADVS-12-2501434-s001.docx]

Supplementary Information:

## Comprehensive Study of Li Deposition and Solid Electrolyte Cracking by Integrating Simulation and Experimental Data

## 1. Note 1: Methodology in details

### 1.1. Constitutive relationship considering large deformation

As estimated by Gao *et al*. ^[1]^, there are a huge volumetric eigenstrain due to lithium plating that causes a GPa level interface stress and the significant viscoplastic flow of lithium. Thus, different from the previous numerical models, where the deformation is based on the small–strain theory, we develop the model under the large–strain framework and employ a viscoplastic constitutive relationship. The main hypothesis underlying the large strain constitutive theory is to decompose the deformation gradient, **F**, in a multiplicative manner:

$\mathbf{F}=\mathbf{F}^{\text{e}}\mathbf{F}^{*}$, (S1)

where **F**^e^ and **F**^*^ are the deformation gradient caused by elastic and inelastic deformation, respectively. The inelastic deformation gradient tensor, **F**^*^, can be further decomposed as follows:

$\mathbf{F}^{*}=\mathbf{F}^{\text{vp}}\mathbf{F}^{\text{vol}}$, (S2)

where **F**^vp^ is the viscoplasticity of the stressed lithium; **F**^vol^ is the volumetric eigenstrain owing to the increasing fraction of lithium atom inserted interstitially in the host lattice of lithium during plating.

We employ the Norton power law to describe the relationship between viscoplasticity rate and the stress as:

$\mathbf{D}^{\text{vp}}=\left\{\begin{matrix} 0 & \sigma^{\text{eq}}<\sigma_{\text{y}} \\ A\left(\frac{\sigma^{\text{eq}}-\sigma_{\text{y}}}{\sigma_{\text{ref}}^{\text{eq}}}\right)^{n}\frac{\partial\sigma^{\text{eq}}}{\partial\mathbf{\sigma}} & \sigma^{\text{eq}}\geq\sigma_{\text{y}} \end{matrix}\right.$, (S3)

where is the rate of viscoplasticity; *σ*^eq^ and $\sigma_{\text{ref}}^{\text{eq}}$ are the equivalent stress and its reference value, respectively. And In Eq. (S3), *σ*_y_ = *h*_2_*σ*_y, Li_ + (1– *h*_2_)*σ*_y, Li_/*η*, *A* = *h*_2_*A*_Li_ + (1– *h*_2_) *ηA*_Li_, *n* = *h*_2_*n*_Li_ + (1– *h*_2_)*ηn*_Li_ are, respectively, the yield strength, reference viscoplastic rate coefficient and viscoplastic exponent that vary continuously from lithium to other phase with the interpolation function of *h*_2_, where *η* = 10^–9^ is a finite small value ensuring that the viscoplasticity only occurs in the lithium.

Following Ref. ^[2]^, the deformation gradient caused by volumetric eigenstrain, **F**^vol^, can be expressed as:

, (S4)

where $c_{2}^{\text{Li}}$ and are, respectively, the concentration of lithium atom and its reference value; *h*_2_ ensures that volumetric eigenstrain only occurs in the lithium; *J*^vol^ represent the change in volume; and **I** is the second–order identity tensor.

The stress–strain relation is derived based on the entropy imbalance. Ignoring heat transfer, the entropy imbalance can be expressed as:

$\frac{\partial W^{\text{ext}}}{\partial t}-\frac{\partial\psi^{\text{mech}}}{\partial t}=\frac{\partial S}{\partial t}T\geq0$, (S5)

where $W^{\text{ext}}$, $\psi^{\text{mech}}$ and $S$ are the external power density, elastic strain energy density per unit volume of the initial state, and entropy density, respectively; and *T* is the thermodynamic temperature. And the external power density and the elastic strain energy density are, respectively, expressed as:

$W^{\text{ext}}=\mathbf{P}:\mathbf{F}=\mathbf{P}:\left(\mathbf{F}^{\text{e}}\mathbf{F}^{\text{vp}}\mathbf{F}^{\text{vol}}\right)$ and (S6)

$\psi^{\text{mech}}=J^{\text{vol}}\psi_{0}^{\text{mech}}=J^{\text{vol}}\left(\frac{1}{2}\mathbf{E}^{\text{e}}:\mathbf{C}^{\text{e}}:\mathbf{E}^{\text{e}}\right)$, (S7)

where **P** is the first Piola–Kirchhoff stress tensor; $\psi_{0}^{\text{mech}}$ is the elastic strain energy per unit volume of the intermediate state, which is converted to $\psi^{\text{mech}}$ by multiplying the rate of volumetric change between intermediate and initial state, *J*^vol^;  is the Green–Lagrange elastic strain tensor; $\mathbf{C}^{\text{e}}$ is the fourth–order elastic stiffness tensor; and the symbol ‘:’ is the internal product between two tensor defined as $\mathbf{E}^{\text{e}}:\mathbf{C}^{\text{e}}:\mathbf{E}^{\text{e}}=E_{ij}^{\text{e}}C_{ijkl}^{\text{e}}E_{kl}^{\text{e}}$.

During plating, if friction is ignored, the internal stress is only the result of the vertical extrusion of deposited lithium against the wall surface of SEs (or anode). Therefore, the elastic stiffness tensor is split into two parts:

$\mathbf{C}^{\text{e}}=\mathbf{C}_{\text{bulk}}^{\text{e}}+\mathbf{C}_{\text{contact}}^{\text{e}}$, (S8)

where $\mathbf{C}_{\text{bulk}}^{\text{e}}$represents the bulk stiffness; and $\mathbf{C}_{\text{contact}}^{\text{e}}$ denotes the stiffness along the tangential direction of the surface of deposited lithium. They are, respectively, expressed as:

$\mathbf{C}_{\text{bulk}}^{\text{e}}=h_{1}\left(\left(1-\chi\left(\theta\right)\right)+\eta\chi\left(\theta\right)\right)\mathbf{C}_{1}^{\text{e}}+h_{2}\mathbf{C}_{2}^{\text{e}}+h_{3}\mathbf{C}_{3}^{\text{e}}+\eta h_{4}\mathbf{C}_{1}^{\text{e}}$ and (S9)

$\mathbf{C}_{\text{contact}}^{\text{e}}=16\left(p_{2}\right)^{2}\left(1-p_{2}\right)^{2}\mathbf{T}\left(p_{2}\right)\eta\mathbf{C}_{1}^{\text{e}}=16\left(p_{2}\right)^{2}\left(1-p_{2}\right)^{2}\left(\mathbf{I}-\frac{\nabla p_{2}}{\left|\nabla p_{2}\right|}\otimes\frac{\nabla p_{2}}{\left|\nabla p_{2}\right|}\right)\eta\mathbf{C}_{1}^{\text{e}}$, (S10)

where $\mathbf{C}_{1}^{\text{e}}$, $\mathbf{C}_{2}^{\text{e}}$, $\mathbf{C}_{3}^{\text{e}}$ are respectively, the stiffness matrix of SEs, deposited lithium and metallic anode, which are in combination with the interpolation functions of *h_α_* and *χ*(*θ*) to mollify the material discontinuity and consider the effect of crack; 16(*p*_2_)^2^(1–*p*_2_)^2^ ensures that the stiffness matrix, $\mathbf{C}_{\text{contact}}^{\text{e}}$, is only significant at the surface of deposited lithium; **T**(*p*_2_) is the tangential directional tensor of the surface of deposited lithium; and *η* is to give the crack, the gap between SEs and anode, and the tangential direction of the surface of deposited lithium a very small stiffness to eliminate stress.

Substituting Eq. (S6, S7) into Eq (S5) and using the relation involving the internal product of tensors, **A** : (**BC**) = (**AC***^T^*): **B**, the entropy imbalance is expressed as:

$\mathbf{P}:\left(\mathbf{F}^{\text{e}}\frac{\partial\mathbf{F}^{\text{vp}}}{\partial t}\mathbf{F}^{\text{vol}}+\mathbf{F}^{\text{e}}\mathbf{F}^{\text{vp}}\frac{\partial\mathbf{F}^{\text{vol}}}{\partial t}\right)-\left(\frac{\partial\psi^{\text{mech}}}{\partial\mathbf{F}^{\text{e}}}-\mathbf{P}\left(\mathbf{F}^{\text{vol}}\right)^{T}\left(\mathbf{F}^{\text{vp}}\right)^{T}\right):\frac{\partial\mathbf{F}^{\text{e}}}{\partial t}\geq0$. (S11)

With the above thermodynamic inequality and following the Onsager principle, the first Piola–Kirchhoff stress can be derived as:

$\mathbf{P}=\frac{\partial\Psi^{\text{mech}}}{\partial\mathbf{F}^{\text{e}}}\left(\left(\mathbf{F}^{\text{vol}}\right)^{T}\left(\mathbf{F}^{\text{vp}}\right)^{T}\right)^{-1}=\mathbf{F}^{\text{e}}\frac{\partial\Psi^{\text{mech}}}{\partial\mathbf{E}^{\text{e}}}\left(\left(\mathbf{F}^{\text{vol}}\right)^{T}\left(\mathbf{F}^{\text{vp}}\right)^{T}\right)^{-1}$. (S12)

According to Neto *et al*. ^[3]^, the first Piola–Kirchhoff stress tensor, **P**, can be expressed in the form of Cauchy stress tensor, **σ**, as **σ** = det(**F**)^–1^**PF***^T^*, where det(•) means the determinant of a matrix. Thus, Eq. (S12) finally leads to:

$\mathbf{\sigma}=\det\left(\mathbf{F}\right)^{-1}\left(\mathbf{F}^{\text{e}}\frac{\partial\Psi^{\text{mech}}}{\partial\mathbf{E}^{\text{e}}}\left(\left(\mathbf{F}^{\text{vol}}\right)^{T}\left(\mathbf{F}^{\text{vp}}\right)^{T}\right)^{-1}\right)\mathbf{F}^{T}$, (S13)

which is constitutive relationship under the large–strain framework to characterize the deformation of lithium–SEs systems during plating.

### 1.2. Helmholtz free energy for the lithium–SEs–anode system

Considering the mechanical, interfacial, chemical, and electric contributions, the total Helmholtz free energy of the system, Ψ, can be split into four parts:

$\Psi=\int_{\Omega} \left(\psi^{\text{mech}}+\psi^{\mathrm{int}}+\psi^{\text{chem}}+\psi^{\text{elec}}\right)d\omega$, (S14)

where *ψ*^int^, *ψ*^chem^, *ψ*^elec^ are the interfacial, chemical, and electropotential energy densities, respectively.

Following Chatterjee *et al*. ^[4]^, the interfacial energy density is constructed as:

$\psi^{\mathrm{int}}\left(\mathbf{p}\right)=\frac{1}{2}\kappa\sum_{i=1}^{N} \left(\nabla p_{i}\right)^{2}+m\left(\sum_{i=1}^{N} \left(\frac{1}{4}\left(p_{i}\right)^{4}-\frac{1}{2}\left(p_{i}\right)^{2}\right)+\sum_{i=1}^{N} \sum_{j=1,j\neq i}^{N} \left(\frac{3}{4}\left(p_{i}p_{j}\right)^{2}\right)\right)$, (S15)

where the first term of the right–hand side is the gradient term to form a the diffuse–interface with a certain width; the second term is the barrier function, which is analog to the fourth–order Landau polynomial potential to ensure that the different phases are separated; *κ* and *m* are the coefficient of gradient energy density and the parameter to scale the height of the energy barrier, which can be related to the interfacial energy density (per unit area), *s*, and the interfacial thickness, *δ*, expressed as *κ* = (3/4)*sδ* and *m* = 6(*s*/*δ*), respectively.

Following Kim *et al.* ^[5]^, the chemical free energy density is construct to be the mixture of the free energy density of *α* (= 1, 2, 3, 4) phase, $\psi_{\alpha}^{\text{chem}}$:

$\psi^{\text{chem}}=\sum_{\alpha=1}^{N} h_{\alpha}\psi_{\alpha}^{\text{chem}}\left(c_{\alpha}^{\text{Li}},\text{ }c_{\alpha}^{\text{Li}^{+}}\right)$, (S16)

where the superscripts, Li, Li^+^ represent the lithium atoms and ions, respectively; subscripts, *α* (= 1, 2, 3, 4), represent different phases for SEs (*α* = 1), deposited lithium (*α* = 2), metallic anode (*α* = 3) and the gap between SEs and anode (*α* = 4), respectively. $c_{\alpha}^{*}$, in Eq. (S16) is the phase concentration of component ⁎ in *α* phase, which is a fictitious concentration, having the following relationship with the actual concentration of component, *c*^⁎^ ^[5]^:

$c^{*}=\sum_{\alpha=1}^{N} h_{\alpha}c_{\alpha}^{*}$. (S17)

$\psi_{\alpha k}^{\text{chem}}$ can be generalized in the form of rectangular–well potential based on the actual chemical potential data. For simplicity, $\psi_{\alpha}^{\text{chem}}\left(c_{\alpha}^{\text{Li}},\text{ }c_{\alpha}^{\text{Li}^{+}}\right)$, in present model is expressed as ideal solution:

, (S18)

where is the reference concentration of component ⁎ in *α* phase; *R* is the ideal gas constant.

The electric potential density, *ψ*^elec^, resulting from the contribution of ions (Li^+^) and electrons (e^–^), is expressed as:

, (S19)

where *φ*_SE_, *φ*_Anode_ are the electric potential of SEs and anode, respectively; and $c^{\text{e}^{\text{-}}}$is the concentration of electrons.

### 1.3. Reaction kinetics for lithium plating

Following Bazant ^[6]^, the reaction rate, *r*, can be expressed as:

$r=k^{0}\left(a_{\text{R}}\exp\left(\frac{(1-\rho)\left(\mu_{\text{R}}^{\text{ex}}-\mu_{\text{P}}^{\text{ex}}\right)}{RT}\right)-a_{\text{P}}\exp\left(-\frac{\rho\left(\mu_{\text{R}}^{\text{ex}}-\mu_{\text{P}}^{\text{ex}}\right)}{RT}\right)\right)$, (S20)

where *k*_0_ is the kinetics constant; *ρ* is the asymmetric parameter; *a*_R_ and *a*_P_ are, respectively, the activity of reactants and products; and $\mu_{\text{R}}^{\text{ex}}-\mu_{\text{P}}^{\text{ex}}$ is the difference of excess chemical potential between reactant and products.

Considering the reaction Li^+^ + e^–^ → Li, *a*_R_ and *a*_P_ are, respectively, expressed as:

$a_{\text{R}}=a_{1}^{\text{Li}^{\text{+}}}a^{\text{e}^{\text{-}}}$. (S21)

$a_{\text{P}}=a_{2}^{\text{Li}}$. (S22)

where the $a_{1}^{\text{Li}^{\text{+}}}$and $a_{2}^{\text{Li}}$are, respectively, the activity of lithium ions in SEs and the activity of atoms in deposited lithium, respectively; and $a^{\text{e}^{\text{-}}}$is the activity of electron. According to the definition of Bazant ^[6]^, the activity of component ⁎ is concentration dependent, given by:

$a_{\alpha}^{k}=\exp\left(\frac{\partial\psi^{\text{chem}}}{RT\partial c_{\alpha}^{*}}\right)$. (S23)

Following the quasi–equilibrium condition of KKS model, the partial derivative of *ψ*^chem^ to $c_{\alpha}^{*}$ can be expressed as:

$\frac{\partial\psi^{\text{chem}}}{\partial c_{1}^{*}}=\ldots=\frac{\partial\psi^{\text{chem}}}{\partial c_{\alpha}^{*}}=\ldots=\frac{\partial\psi^{\text{chem}}}{\partial c_{N}^{*}}=\mu_{\text{chem}}^{*}$. (S24)

Thus, the activity of component Li^+^ in SE, $a_{1}^{\text{Li}^{\text{+}}}$, and Li atom in lithium, $a_{2}^{\text{Li}}$, are, respectively, expressed as:

$a_{1}^{\text{Li}^{\text{+}}}=\exp\left(\frac{\mu_{\text{chem}}^{\text{Li}^{\text{+}}}}{RT}\right)$. (S25)

$a_{2}^{\text{Li}}=\exp\left(\frac{\mu_{\text{chem}}^{\text{Li}}}{RT}\right)$. (S26)

And since the transportation of electron in lithium is super–fast, the activity of component e^–^, $a^{\text{e}^{\text{-}}}$, is set to be unit.

And the difference of excess chemical potential between reactant and products, $\mu_{\text{R}}^{\text{ex}}-\mu_{\text{P}}^{\text{ex}}$, is expressed as

. (S27)

where is the excess chemical potential of electron; and and are, respectively, the excess chemical potential of Li ion in SE and Li atom in lithium. Following Bazant ^[6]^, the excess chemical potential involves the contributions of electric field and mechanical deformation. Therefore, , and are, respectively, expressed as:

, (S28)

, (S29)

. (S30)

Eq. (S30) indicates a mechano–electrochemical coupling during deposition reaction. In Eq. (S30), the derivative of elastic strain energy density, $\Psi^{\text{mech}}$, with respect to $\mathbf{F}^{\text{e}}$ can be expressed in the form Cauchy stress tensor, **σ**, i.e.,

. (S31)

The derivative of tensor, $\partial\mathbf{F}^{\text{e}}/\partial\mathbf{F}^{\text{*}}$, can be expressed in Cartesian component form as:

$\frac{\partial\mathbf{F}^{\text{e}}}{\partial\mathbf{F}^{*}}=\left(\frac{\partial F_{\text{mn}}^{\text{e}}}{\partial F_{iJ}^{*}}\right)=\left(\frac{\partial\left(F_{mK}f_{Kn}^{*}\right)}{\partial F_{iJ}^{*}}\right)=\left(F_{mK}\frac{\partial f_{Kn}^{*}}{\partial F_{iJ}^{*}}\right)$, (S32)

where $F_{mn}^{\text{e}}$, $F_{\text{mK}(\text{or iJ})}^{*}$ and $f_{\text{Kn}}^{*}$ are the components of elastic gradient tensor, $\mathbf{F}^{\text{e}}$, inelastic gradient tensor, $\mathbf{F}^{*}$, and its inverse , respectively. Using the relation of derivative of the inverse of a tensor ^[3]^, the Cartesian component, $\partial f_{Kn}^{*}/\partial F_{iJ}^{*}$, is expressed as:

$\frac{\partial f_{Kn}^{*}}{\partial F_{iJ}^{*}}=-f_{Ki}^{*}f_{Jn}^{*}$. (S33)

Substituting Eq. (S33) into Eq. (S32), the derivative of tensor, $\partial\mathbf{F}^{\text{e}}/\partial\mathbf{F}^{\text{*}}$, can be rewritten as:

. (S34)

The derivative of $J^{\text{vol}}$ with respect to $c_{2}^{\text{Li}}$, in Eq. (S30), can be expressed as:

$\frac{\partial J^{\text{vol}}}{\partial c_{2}^{\text{Li}}}=\frac{\partial J^{\text{vol}}}{\partial\mathbf{F}^{*}}\frac{\partial\mathbf{F}^{*}}{\partial c_{2}^{\text{Li}}}$. (S35)

Using the relation ^[3]^:

, (S36)

Eq. (S35) is recast as:

. (S37)

Substituting Equations. (S31, S34, S37) into Equation (S30) leads to the final form of the excess chemical potentials, , expressed as:

. (S38)

Eq. (S38) indicates that the stress–dependent chemical potential, , involves the effects of conformational volume change and deformation energy. If the small deformation assumption is adopted, the effects of conformational volume change and deformation energy would be insignificant, leading to:

. (S39)

### 1.4. Governing equations

#### 1.4.1 Lithium deposition under weak or no mechanical constraint

According to the experimental results by Gao *et al*. ^[1]^, if the Li deposition is under a weak or no mechanical constraint condition, the so–called root–growth mechanism would be significant. In this scenario, the newly formed Li atoms deposit onto the reaction interface, which push away the original host lattice. It leads to the following governing equation of order parameter field:

$\left\{\begin{matrix} \frac{\partial p_{1}}{\partial t}=-M\left(-\kappa\nabla^{2}p_{1}+m\left(\left(p_{1}\right)^{3}-p_{1}+3p_{2}\sum_{j\neq1}^{N} \left(p_{j}\right)^{2}\right)\right) \\ \frac{\partial p_{2}}{\partial t}=-M\left(-\kappa\nabla^{2}p_{2}+m\left(\left(p_{2}\right)^{3}-p_{2}+3p_{2}\sum_{j\neq2}^{N} \left(p_{j}\right)^{2}\right)\right)+4h_{2}h_{3}\frac{r}{c_{\text{ref}}^{\text{Li}}} \\ \frac{\partial p_{3}}{\partial t}=-M\left(-\kappa\nabla^{2}p_{3}+m\left(\left(p_{3}\right)^{3}-p_{3}+3p_{3}\sum_{j\neq3}^{N} \left(p_{j}\right)^{2}\right)\right)-4h_{3}\left(h_{2}+h_{4}\right)\frac{r}{c_{\text{ref}}^{\text{Li}}} \\ \frac{\partial p_{4}}{\partial t}=-M\left(-\kappa\nabla^{2}p_{4}+m\left(\left(p_{4}\right)^{3}-p_{4}+3p_{4}\sum_{j\neq4}^{N} \left(p_{j}\right)^{2}\right)\right)+4h_{3}h_{4}\frac{r}{c_{\text{ref}}^{\text{Li}}} \\ \left(4h_{2}h_{3}\bar{\sigma}_{\text{mises}}\leq\sigma_{\text{y}}^{\text{Li}}\right) \end{matrix}\right.$, (S40)

where $4h_{2}h_{3}r/c_{\text{ref}}^{\text{Li}}$ is the driving force for the evolution of Li, which is mediated by reaction kinetics; $4h_{3}\left(h_{2}+h_{4}\right)r/c_{\text{ref}}^{\text{Li}}$ characterizes the rigid body movement of flexible CC; and $4h_{3}h_{4}r/c_{\text{ref}}^{\text{Li}}$ decribes the widening of gap between SEs and CC; $4h_{2}h_{3}\bar{\sigma}_{\text{mises}}$ denotes the average contact mises stress along Li|CC interface; $4h_{2}h_{3}\bar{\sigma}_{\text{mises}}\leq\sigma_{\text{y}}^{\text{Li}}$ indicates that the root–growth only occurs when the average contact mises stress is smaller than the yield strength of Li. Since the deposited Li atoms do not interstitially insert into host lattice, it leads $\mu_{\text{R}}^{\text{ex}}-\mu_{\text{P}}^{\text{ex}}$ to be , where $4h_{1}h_{2}\mathbf{\sigma}$ is the contact stress between SEs and lithuim.

According to the mass conservation laws for Li ions in SEs, it leads to the reaction diffusion equation of lithium ions, as follows:

$\frac{\partial c^{\text{Li}^{\text{+}}}}{\partial t}=-\nabla\mathbf{j}^{\text{Li}^{\text{+}}}-r$. (S41)

where $\mathbf{j}^{\text{Li}^{\text{+}}}$and –*r* are, respectively, the flux and reaction rate of Li ions. Considering Eq. (S19) and regarding $\mu_{\text{chem}}^{\text{Li}^{+}}$ and {*p*_1_, …, *p_α_*, …, *p_N_*} as the functions of $c^{\text{Li}^{\text{+}}}$, the temporal evolution of a component Li ions can be expressed as:

$\frac{\partial c^{\text{Li}^{\text{+}}}}{\partial t}=\sum_{\alpha=1}^{N} \frac{\partial h_{\alpha}}{\partial t}c_{\alpha}^{\text{Li}^{\text{+}}}+\sum_{\alpha=1}^{N} h_{\alpha}\frac{\partial c_{\alpha}^{\text{Li}^{\text{+}}}}{\partial\mu_{\text{chem}}^{\text{Li}^{+}}}\frac{\partial\mu_{\text{chem}}^{\text{Li}^{+}}}{\partial t}$. (S42)

Based on the Onsager linear law, the flux, $\mathbf{j}^{\text{Li}^{\text{+}}}$, is expressed as:

, (S43)

indicating that the diffusion of lithium ion is driven by the gradient of chemical and electric potential in SEs. $\mathbf{D}_{\text{Li}^{\text{+}}}^{\text{diff}}$ in Eq. (S43), is the diffusion matrix of lithium ion. Considering the fast diffusion–pathways along interface and surface, $\mathbf{D}_{\text{Li}^{\text{+}}}^{\text{diff}}$, is expressed as:

, (S44)

where , and are, respectively, the diffusive matrix of lithium ion in the body of SEs, along the interface of SEs–lithium and SEs–anode, and along the surface of SE. They are, respectively, expressed as:

, (S45)

, (S46)

, (S47)

where *η* ensures that transportation of Li ions occurs only in SEs; 4(*h*_1_*h*_2_+*h*_1_*h*_3_) and 4(*h*_1_*h*_4_+*h*_1_*χ*(*θ*)) is to consider the diffusivity of Li ions along the interface of SEs–lithium and SEs–anode, and along the surface of SE, respectively; **T**(*p*_1_) is the tangential directional tensor of the surface of SEs. Substituting Eq. (S42, S43) into Eq. (S41), we have:

. (S48)

The Poisson’s equation is employed to govern the electropotential field, *φ*_SE_, given by:

, (S49)

where *ε* = *ε*_S_(*h*_1_(1–*χ*(*θ*))+*h*_3_) + *ε*_SE_*h*_2_ + *η*(*h*_1_*χ*(*θ*)+*h*_4_) is the effective electric conductivity of the medium with *ε*_S_ and *ε*_SE_ being the conductivities of the conductor and SEs; *η* is to ensure that the unfilled gap and crack are electrically insulated.

#### 1.4.2 Lithium deposition under strong mechanical constraint

If the lithium deposition is under a strong mechanical constraint, the filling of lithium in the crack or the gap via viscoplastic flow would be significant, leading to the governing equation of order parameter field, as follows:

$\frac{\partial p_{i}}{\partial t}=-M\left(\frac{\partial\psi^{\mathrm{int}}}{\partial p_{i}}+\frac{\partial\psi^{\text{chem}}}{\partial p_{i}}\right)\text{ }\left(4h_{2}\left(1-h_{2}\right)\bar{\sigma}_{\text{mises}}>\sigma_{\text{y}}^{\text{Li}}\text{ }\right)$, (S50)

where $4h_{2}\left(1-h_{2}\right)\bar{\sigma}_{\text{mises}}$ represents the average contact stress between the Li and other phase; $4h_{2}\left(1-h_{2}\right)\bar{\sigma}_{\text{mises}}>\sigma_{\text{y}}^{\text{Li}}$ indicates that viscoplastic flow only occurs when the average contact stress is larger than the yield strength of Li; *M* is the scalar mobility for phase transformation. It is expressed as:

$M=\frac{\sum_{i=1}^{N} \sum_{j=1,j\neq i}^{N} M_{ij}\left(p_{i}p_{j}\right)^{2}}{\sum_{i=1}^{N} \sum_{j=1,j\neq i}^{N} \left(p_{i}p_{j}\right)^{2}}$, (S51)

where *M_ij_* is the mobility of interface between *i* and *j* phases. Especially, *M*_12_ = *M*_12_(*χ*(*θ*) + *η*(1–*χ*(*θ*))) is to ensure that the deposited lithium can freely fill the crack rather than penetrate into SEs.

Substituting Eq. (S15, S16) into Eq. (S50), the governing equation of order parameter field can be further expressed as:

$\left\{\begin{matrix} \frac{\partial p_{i}}{\partial t}=-M_{\eta}\left(\begin{matrix} -\kappa\nabla^{2}p_{i}+m\left(\left(p_{i}\right)^{3}-p_{i}+3p_{i}\sum_{j\neq i}^{N} \left(p_{j}\right)^{2}\right)+ \\ \sum_{\alpha=1}^{N} \frac{\partial h_{\alpha}}{\partial p_{i}}\psi_{\alpha}^{\text{chem}}+\sum_{\alpha=1}^{N} h_{\alpha}\left(\frac{\partial\psi_{\alpha}^{\text{chem}}}{\partial c_{\alpha}^{\text{Li}}}\frac{\partial c_{\alpha}^{\text{Li}}}{\partial p_{i}}+\frac{\partial\psi_{\alpha}^{\text{chem}}}{\partial c_{\alpha}^{\text{Li}^{+}}}\frac{\partial c_{\alpha}^{\text{Li}^{+}}}{\partial p_{i}}\right) \end{matrix}\right) \\ \left(4h_{2}\left(1-h_{2}\right)\bar{\sigma}_{\text{mises}}>\sigma_{\text{y}}^{\text{Li}}\text{ }\right) \end{matrix}\right.$. (S52)

According to KKS model ^[5]^, the decoupling between order parameter, **p**, and the actual concentration of component, *c*^⁎^, can be achieved, leading to:

$\frac{\partial c^{*}}{\partial p_{i}}=\frac{\partial\sum_{\alpha=1}^{N} h_{\alpha}c_{\alpha}^{*}}{\partial p_{i}}=\sum_{\alpha=1}^{N} \frac{\partial h_{\alpha}}{\partial p_{i}}c_{\alpha}^{*}+\left(\sum_{\alpha=1}^{N} h_{\alpha}\frac{\partial c_{\alpha}^{*}}{\partial\mu^{*}}\right)\frac{\partial\mu^{*}}{\partial p_{i}}\equiv0$. (S53)

Thus, $\partial c_{\alpha}^{*}/\partial p_{i}$, in Eq. (S52), can be expressed as:

$\frac{\partial c_{\alpha}^{*}}{\partial p_{i}}=\frac{\partial c_{\alpha}^{*}}{\partial\mu^{*}}\frac{\partial\mu^{*}}{\partial p_{i}}=-\frac{\sum_{\beta=1}^{N} \frac{\partial h_{\beta}}{\partial p_{i}}c_{\beta}^{*}}{\sum_{\beta=1}^{N} h_{\beta}\frac{\partial c_{\beta}^{*}}{\partial\mu^{*}}}\frac{\partial c_{\alpha}^{*}}{\partial\mu^{*}}$. (S54)

Substituting Eqs. (S54) into Eq. (S52) and considering the quasi–equilibrium condition (Eq. (S24)), the governing equation of order parameter field can be finally expressed as:

$\left\{\begin{matrix} \frac{\partial p_{i}}{\partial t}=-M_{\eta}\left(\begin{matrix} -\kappa\nabla^{2}p_{i}+m\left(\left(p_{i}\right)^{3}-p_{i}+3p_{i}\sum_{j\neq i}^{N} \left(p_{j}\right)^{2}\right)+ \\ \sum_{\alpha=1}^{N} \frac{\partial h_{\alpha}}{\partial p_{i}}\left(\psi_{\alpha}^{\text{chem}}-\mu^{\text{Li}}c_{\alpha}^{\text{Li}}-\mu^{\text{Li}^{+}}c_{\alpha}^{\text{Li}^{+}}\right) \end{matrix}\right) \\ \left(4h_{2}\left(1-h_{2}\right)\bar{\sigma}_{\text{mises}}>\sigma_{\text{y}}^{\text{Li}}\text{ }\right) \end{matrix}\right.$, (S55)

where $\psi_{\alpha}^{\text{chem}}-\mu^{\text{Li}}c_{\alpha}^{\text{Li}}-\mu^{\text{Li}^{+}}c_{\alpha}^{\text{Li}^{+}}$is driving force for the evolution of order parameter filed.

Since variation of driving force is owing to the diffusion and reaction of Li ions and atoms, it leads to the reaction diffusion equation of Li atoms, as follows:

,

(S56)

where $\mathbf{D}_{\text{Li}}^{\text{diff}}$ is the diffusion matrix of lithium atom. It is expressed as:

, (S57)

, (S58)

, (S59)

, (S60)

where , and are, respectively, the diffusive matrix of lithium atom in the host lattice, along the interface of lithium–SEs and lithium–anode, and along the surface of lithium; **T**(*p*_2_) is the tangential directional tensor of the surface of lithium.

Eq. (S56) indicates that the diffusion of lithium atoms is controlled by gradient of chemical potential and the stress. Such a gradient is owing to the increasing fraction of lithium atom inserted interstitially in the host lattice. Thus, the diffusion of lithium atoms, decreasing the fraction of inserted atom, results in a localized relaxation of stress and a flow of lithium to fill the crack and gap, which is consistent with the mechanism of atomic diffusion–based viscoplasticity.

Based on the fracture PF theory, the governing equation of cracking in SEs can be expressed as ^[7]^:

$\frac{G_{\text{f}}}{l}(\theta-l^{2}\nabla^{2}\theta)=2(1-\theta) Yh_{1}$, (S61)

where $G_{\mathrm{f}}$ is the energy dissipated upon the creation of a unit on the fracture surface; *l* denotes the thickness of the interface between the SEs and crack, and it can be regarded as a pure numerical parameter; *Y* is the effective crack driving force; and *h*_1_ ensures the cracking only occur in SEs. For the cracking of brittle material, *Y* is expressed as:

$Y=\frac{\left(\sigma_{1}\right)^{2}}{2E_{\text{SE}}}$, (S62)

where *σ*_1_ and *E*_SE_ are, respectively, the first principal stress and the Young’s modulus of SEs. To ensure the crack irreversibility, a numerical approach is to replace *Y* with its maximum value of $H=\text{max}\left(Y,\max_{\left(0,t_{n}\right)} \left(Y_{t_{n}}\right)\right)$ ^[8]^, where *t_n_* represents the *n*th time–step in simulation, and $Y_{t_{n}}$ is the effective crack driving force of the *n*th time–step. It indicates that when $Y\leq\max_{\left(0,t_{n}\right)} \left(Y_{t_{n}}\right)$, the material remains its elasticity and does not crack.

Thus, by coupling the constitutive relationship (Eq. S13), the diffusion reaction equation of lithium ions and atoms (Eq. (S48, S56)), the Poisson’s equation (Eq. (S49)) and the governing equation of order parameter field (Eq. (S55)) and fracture (Eq. (S61)), the cracking of SEs and the filling of lithium in the crack or the gap via viscoplastic flow under a strong or rigid constraint are characterized.

## 3. Note 2: Material parameters in simulation

In present simulation, the temperature in simulation is set at 293K. The Newton–Raphson method is employed to solve the nonlinear governing equations, in which the time–step is automatically refined to ensure the convergence of the solution step. In present simulations, the initial and maximum time steps are, respectively, 10^–6^ *t*_ref_ and 10^–3^ *t*_ref_ for temporal integration, where *t*_ref_ is the reference time. The parameters used in simulation are listed in Table S1.

Table S1

|  | Parameter | Value |
| --- | --- | --- |
| Reference length | *l*_ref_ | 1 μm |
| Reference time | *t*_ref_ | 1 s |
| Interface thickness | *δ* | 0.02 μm |
| interfacial energy density | *s* | 0.1 J m^–2^ |
| Young’s modulus of the Li | *E*_Li_ | 4.9 GPa ^[9]^ |
| Young’s modulus of the SEs | *E*_SE_ | 60–140 GPa ^[9]^ |
| Poisson’s ratio of the Li | *v*_Li_ | 0.4 ^[9]^ |
| Poisson’s ratio of the SE | *v*_SE_ | 0.257 ^[9]^ |
| Stress exponent of Li for viscoplasticity | *n* | 6.6 ^[10]^ |
| Viscoplastic rate coefficient of Li | *A*^–1/n^ | 3×10^–5^ Pa s^–1^ ^[10]^ |
| Yield strength of Li | $\sigma_{\text{y}}^{\text{Li}}$ | 14 MPa ^[1]^ |
| Reference concentration of Li | $c_{\text{Li,S}}^{\text{ref}}$ | 76.4 mol L^–1^  ^[11]^ |
| Reference concentration of Li ion in SE | $c_{\text{Li}^{+}\text{,SE}}^{\text{ref}}$ | 4.22 mol L^–1^  ^[12]^ |
| Asymmetry factor | *ρ* | 0.05 |
| Diffusion coefficient of lithium atom in the body of metallic Lithium |  | 1 ×10^–15^ m^2^ s^–1^ ^[13]^ |
| Diffusion coefficient of lithium atom along interface |  | 1 ×10^–13^ m^2^ s^–1^ ^[13]^ |
| Diffusion coefficient of lithium atom along interface surface |  | 1 ×10^–11^ m^2^ s^–1^ ^[13]^ |
| Diffusion coefficient of lithium ion in the body of SEs |  | 1 ×10^–12^ m^2^ s^–1^ ^[14]^ |
| Diffusion coefficient of lithium atom along interface |  | 1 ×10^–10^ m^2^ s^–1^ |
| Diffusion coefficient of lithium atom along interface surface |  | 1 ×10^–8^ m^2^ s^–1^ |
| Electric conductivity of SE | *ε*_SE_ | 1 S m^–1^ ^[15]^ |
| Electric conductivity of Li deposit | *ε*_S_ | 10^7^ S m^–1^ ^[15]^ |
| Fracture toughness of SE | *K*_IC_ | 0.95 MPa m^0.5^ ^[16]^ |
| Geometric factor for fracture | *Y* | 1.12 ^[17]^ |
| Energy dissipated upon the creation of a unit on the fracture surface (fracture energy density) | *G*_f_ | 15–25 J m^2^ |
| Thickness of the Crack|SEs interface | *l* | 0.001 μm |
| Ideal gas constant | *R* | 8.314 J mol^–1^ K^–1^ |
| Faraday’s constant | *F* | 96485 C mol^–1^ |

## Note 3 Boundary conditions and Supplementary results

### Note 3.1. Low electric-potential plating with variable constraints

Since the chemical potential field rather than the concentration field is solved based on Eq. (S48 and S56), the initial and boundary conditions should be given in the form of chemical potential. We assume that the deposits and SE are initially at a state of chemical equilibrium, therefore $\mu^{\text{Li}^{+}}$= 0 and $\mu^{\text{Li}}$= 0 at *t* = 0. Also, chemical equilibrium, $\mu^{\text{Li}^{+}}$= 0 and $\mu^{\text{Li}}$= 0, are imposed as the Dirichlet boundary conditions on the up and bottom sides of the simulation domain. For the other sides, the zero-flux boundary conditions, $\nabla\mu^{\text{Li}^{+}}$= 0 and $\nabla\mu^{\text{Li}}$= 0, are applied. As shown in Fig. S1(a), for the electrical potential, the Dirichlet boundary conditions *φ* = 0 and *φ* = *φ*_apply_ are set at the upper surface of the CC and the bottom surface of SE, respectively; the zero-flux condition, ∇*φ* = 0, is applied to the other sides. For mechanical deformation, stack stress, *σ*_sp_, related to the elongation of the Li whisker, ∆*l*, was applied to the upper surface of the CC and expressed as *σ*_sp_ = *k*_s_∆*l*, where *k*_s_ is the spring constant of the probe cantilever (*k*_s_ = 5 N/m in the experiment ^[1]^); the left and bottom side are constrained along their normal direction; the other sides are free.





Fig. S1 (a) the initial distribution of order parameter field, **p**, the geometry size of domain and the boundary condition for low electric-potential plating with variable constraints, and (b) the corresponding finite element mesh with a maximum size of 0.04 μm.





Fig. S2 Schematic diagram of the transition from (a) root–growth to (b) shank–expansion for the low electric-potential plating scenario with variable constraints; and (c) the stress evolution of mises stress in Li during plating

### Note 3.2. high electric-potential plating with ridge constraints

In this scenario, the initial and boundary conditions of chemical potential are the same as the scenario for low electric-potential plating with variable constraints. For electrical potential, the Dirichlet boundary conditions *φ* = 0 and *φ* = *φ*_apply_ are set at the bottom and up sides of simulation domain, respectively; the zero-flux condition, ∇*φ* = 0, is applied to the other sides (See Fig. S3(a) and S6(a)). For mechanical deformation, if the SE particle is tightly constrained by other particles, all of the sides are constrained along their normal direction (see Fig. S3(a)); if the SE particle is isolated, the left and bottom sides are constrained along their normal direction, while the other sides are free (see Fig. S6(a)).

#### Note 3.2.1 Particles tightly constrained by other particles





Fig. S3 (a) the initial distribution of order parameter field, **p**, the geometry size of domain and the boundary condition for high electric-potential plating with ridge constraints; and (b) the corresponding finite element mesh with the minimum size of 0.0004 μm. In this scenario the LLZO particles are tightly constrained by other particles.





Fig. S4 Schematic diagram of plating induced cracking in high electric-potential with ridge constraints for the particles tightly constrained by other particles





Fig. S5 Distribution of (a) electric potential, *φ*, and its gradient of .

#### Note 3.2.2 Isolated SE particles





Fig. S6 (a) the initial distribution of order parameter field, **p**, the geometry size of domain and the boundary condition for high electric-potential plating with ridge constraints; and (b) the corresponding finite element mesh with the minimum size of 0.0004 μm. In this scenario CC is in contact with isolated LLZO particles.





Fig. S7 Schematic diagram of plating induced cracking in high electric-potential with ridge constraints for isolated LLZO particles.

In this scenario, the governing equation of order parameter field, **p**, for the root–growth, is recast as:

$\left\{\begin{matrix} \frac{\partial p_{1}}{\partial t}=-M\left(-\kappa\nabla^{2}p_{1}+m\left(\left(p_{1}\right)^{3}-p_{1}+3p_{2}\sum_{j\neq1}^{N} \left(p_{j}\right)^{2}\right)\right)-4h_{1}\left(h_{2}+h_{4}\right)\frac{r}{c_{\text{ref}}^{\text{Li}}} \\ \frac{\partial p_{2}}{\partial t}=-M\left(-\kappa\nabla^{2}p_{2}+m\left(\left(p_{2}\right)^{3}-p_{2}+3p_{2}\sum_{j\neq2}^{N} \left(p_{j}\right)^{2}\right)\right)+4h_{1}h_{2}\frac{r}{c_{\text{ref}}^{\text{Li}}} \\ \frac{\partial p_{3}}{\partial t}=-M\left(-\kappa\nabla^{2}p_{3}+m\left(\left(p_{3}\right)^{3}-p_{3}+3p_{3}\sum_{j\neq3}^{N} \left(p_{j}\right)^{2}\right)\right) \\ \frac{\partial p_{4}}{\partial t}=-M\left(-\kappa\nabla^{2}p_{4}+m\left(\left(p_{4}\right)^{3}-p_{4}+3p_{4}\sum_{j\neq4}^{N} \left(p_{j}\right)^{2}\right)\right)+4h_{1}h_{4}\frac{r}{c_{\text{ref}}^{\text{Li}}} \\ \left(4h_{1}h_{2}\bar{\sigma}_{\text{mises}}\leq\sigma_{\text{y}}^{\text{Li}}\right) \end{matrix}\right.$, (S63)

where the initial value of order parameter *p*_4_ is set as *χ*(*θ*)(1–*h*_2_) to represent the unfilled crack; $4h_{1}h_{2}r/c_{\text{ref}}^{\text{Li}}$ is the driving force for the evolution of Li, which is mediated by reaction kinetics; $-4h_{1}\left(h_{2}+h_{4}\right)r/c_{\text{ref}}^{\text{Li}}$ characterizes the rigid body movement of sidewall of crack; and $4h_{1}h_{4}r/c_{\text{ref}}^{\text{Li}}$ decribes the widening of crack; $4h_{1}h_{2}\bar{\sigma}_{\text{mises}}$ denotes the average contact mises stress along Li|SEs interface; $4h_{1}h_{2}\bar{\sigma}_{\text{mises}}\leq\sigma_{\text{y}}^{\text{Li}}$ indicates that the root–growth only occurs when the average contact mises stress is smaller than the yield strength of Li.

### Note 3.3. High electric-potential plating without constraints

In this scenario, the initial and boundary conditions of chemical potential are the same as the scenario for low electric-potential plating with variable constraints. For electrical potential, the Dirichlet boundary conditions *φ* = 0 and *φ* = *φ*_apply_ are set at the bottom of CNT and up side of SE, respectively; the zero-flux condition, ∇*φ* = 0, is applied to the other sides (See Fig. S8(a)). For mechanical deformation, the surfaces of SE and the outer wall of CNT are constrained along their normal direction (see Fig. S3(a)), while the other sides are free (see Fig. S8(a))





Fig. S8 (a) the initial distribution of order parameter field, **p**, the geometry size of domain and the boundary condition for high electric-potential plating without constraints, and (b) the corresponding finite element mesh with the minimum size of 0.0004 μm.





Fig. S9 Schematic diagram of high–velocity plating by using CNTs as CC.

**References**

1. H. Gao, X. Ai, H. Wang, W. Li, P. Wei, Y. Cheng, S. Gui, H. Yang, Y. Yang, M.–S. Wang, *Nat. Commun.* **2022**, 13, 5050.
2. Y. N. Zhao, Y. F. Chen, S. G. Ai, D. N. Fang, *Int. J. Plast.* **2019**, 118, 173.
3. E. A. de Souza Neto, D. Peric, D. R. J. Owen, *Computational Methods for Plasticity: Theory and Applications*, John Wiley & Sons Ltd, **2008**.
4. S. Chatterjee, N. Moelans, *Acta Mater.* **2021**, 206, 116630.
5. S. G. Kim, W. T. Kim, T. Suzuki, *Phys. Rev. E* **1998**, 58, 3316.
6. M. Z. Bazant, *Acc. Chem. Res.* **2013**, 46, 1144.
7. M. Ambati, T. Gerasimov, L. De Lorenzis, *Comput. Mech.* **2014**, 55, 383.
8. S. Zhou, X. Zhuang, T. Rabczuk, *Comput. Methods Appl. Mech. Engrg.* **2019**, 350, 169.
9. C. H. Yuan, W. Q. Lu, J. Xu, *Adv. Energy Mater.* **2021**, 2101807.
10. W. S. LePage, Y. X. Chen, E. Kazyak, K. H. Chen, A. J. Sanchez, A. Poli, E. M. Arruda, M. D. Thouless, N. P. Dasgupta, *J. Electrochem. Soc.* **2019**, 166, A89.
11. C. Monroe, J. Newman, *J. Electrochem. Soc.* **2004**, 151, A880.
12. Z. Ahmad, V. Viswanathan, *Phys. Rev. Lett.* **2017**, 119, 056003.
13. Y. Chen, Z. Wang, X. Li, X. Yao, C. Wang, Y. Li, W. Xue, D. Yu, S. Yeon Kim, F. Yang, A. Kushima, G. Zhang, H. Huang, N. Wu, Y. Mai, J. B. Goodenough, J. Li, *Nature* **2020**, 578, 251.
14. K. Tantratian, H. H. Yan, K. Ellwood, E. T. Harrison, L. Chen, *Adv. Energy Mater.* **2021**, 11, 2003417.
15. L. Chen, H. W. Zhang, L. Y. Liang, Z. Liu, Y. Qi, P. Lu, J. Chen, L. Chen, *J. Power Sources* **2015**, 300, 376.
16. G. Han, B. Kinzer, R. Garcia–Mendez, H. Choe, J. Wolfenstine, J. Sakamoto, *J. Eur. Ceram. Soc.* **2020**, 40, 1999.
17. L. Porz, T. Swamy, B. W. Sheldon, D. Rettenwander, T. Frmling, H. L. Thaman, S. Berendts, R. Uecker, W. C. Carter, Y. M. Chiang, *Adv. Energy Mater.* **2017**, 7, 1701003.
